# In vitro generation of genetic diversity for directed evolution by error-prone artificial DNA synthesis

**DOI:** 10.1038/s42003-024-06340-0

**Published:** 2024-05-24

**Authors:** Baowei Wang, Yang Liu, Xuelian Bai, Huijuan Tian, Lina Wang, Miao Feng, Hairong Xia

**Affiliations:** 1 Department of Strategic and Integrative Research, Tianjin Institute of Industrial Biotechnology, Chinese Academy of Sciences, Tianjin, 300308 China; 2 National Center of Technology Innovation for Synthetic Biology, Tianjin, 300308 China; 3 Technique Support and Core Facility Center, Tianjin Institute of Industrial Biotechnology, Chinese Academy of Sciences, Tianjin, 300308 China

**Keywords:** Synthetic biology, Protein design

## Abstract

Generating genetic diversity lies at the heart of directed evolution which has been widely used to engineer genetic parts and gene circuits in synthetic biology. With the ever-expanding application of directed evolution, different approaches of generating genetic diversity are required to enrich the traditional toolbox. Here we show in vitro generation of genetic diversity for directed evolution by error-prone artificial DNA synthesis (epADS). This approach comprises a three-step process which incorporates base errors randomly generated during chemical synthesis of oligonucleotides under specific conditions into the target DNA. Through this method, 200 ~ 4000 folds of diversification in fluorescent strength have been achieved in genes encoding fluorescent proteins. EpADS has also been successfully used to diversify regulatory genetic parts, synthetic gene circuits and even increase microbial tolerance to carbenicillin in a short time period. EpADS would be an alternative tool for directed evolution which may have useful applications in synthetic biology.

## Abstract (web summary)

Error-prone artificial DNA synthesis (epADS) is presented as an effective method for in vitro generation of genetic diversity for directed evolution of various types of genetic parts, synthetic gene circuits and even microbes.

## Introduction

Genetic parts and synthetic gene circuits are primary building blocks in synthetic biology such as cell factories, biosensors, living therapeutics, etc^1–4^. Directed evolution is a powerful technique developed by learning from nature evolution and has been widely used to engineer genetic parts and synthetic gene circuits^5–7^. In general, the implementation of directed evolution includes three steps, generating genetic diversity of the research target, cloning and expression of the genetically diversified DNA into a suitable transcription or translation system, and screening or selection of interested mutants^5^. Generating genetic diversity lies at the heart of directed evolution and can be carried out via semi-rational or random mutagenesis approaches. Semi-rational approaches rely on the understanding of the structure-function relationship of the research target (like proteins and enzymes) and can be carried out via Site-saturation Mutagenesis, Iterative Saturation Mutagenesis, Ancestral Sequence Reconstruction, and other structure-guided methods or computational methods (like PANTHER and PROVEAN)^8–12^. Though semi-rational approaches have shown the advantages of constructing smart libraries with smaller sizes and more focused mutagenesis, random mutagenesis is still appealing to investigators interested in exploring a bigger sequence space. Generating genetic diversity through random mutagenesis may be carried out in vitro or in vivo, which is dependent on the target DNA^5,13,14^. Based on DNA polymerase without proofreading ability (like Taq polymerase), error-prone polymerase chain reaction (ep-PCR) was developed to generate genetic diversity in vitro for directed evolution^15,16^. Despite its convenience, drawbacks including biased mutations towards base transitions and lack of contiguous mutations and indels still exist. Methods including trinucleotide exchange (TriNEx) which randomly substitutes one contiguous trinucleotide sequence for another and random insertional-deletional strand exchange mutagenesis (RAISE) have been developed to deal with these problems^17,18^. In 1994, DNA shuffling, a homologous recombination-based random mutagenesis method was developed for the rapid evolution of a protein in vitro^19^. Since its invention, DNA shuffling has been successfully used in the directed evolution of many proteins, enzymes, and the production of high value-added metabolites^20–23^. Various derivatives of DNA shuffling, such as random chimera genesis on transient templates (RACHITT), nucleotide exchange and excision technology (NExT), and staggered extension process (StEP), have also been reported during this time^24–27^. Although these methods have been successfully implemented in some early studies, they are far from satisfactory. With the ever-expanding application of directed evolution, different procedures with diverse mutation types, higher mutation rates, and ease of execution are required to enrich the toolbox of directed evolution.

Artificial DNA synthesis is one of the foundational technologies in synthetic biology which assembles synthetic oligonucleotides into double-stranded DNA. In 1959, Canadian scientist G. M. Tener and co-workers reported the feasibility of chemical synthesis of oligonucleotides^28^. Six years later, Robert L. Letsinger and V. Mahadevan developed a stepwise method of chemical synthesis of oligonucleotides on solid supports which significantly improved the synthetic efficiency^29^. Thereafter, more efforts were taken to improve the chemical synthesis process which finally led to the commercialization of the chemical synthesis of oligonucleotides and artificial DNA synthesis^30–35^. Since then, artificial DNA synthesis has been used in diverse fields of life science and many important progresses have also been achieved. In 2009, Tian and co-workers reported a high-throughput multiplex DNA synthesis method on a programmable microchip; they also used this method to construct a mutant gene library to optimize protein expression^36,37^. In 2018, Aitao Li and co-workers reported using high-density DNA synthesis on microchips with predesigned oligonucleotides sequences for saturate mutagenesis of specific amino acids of an enzyme; they suggested that DNA synthesis may be the next core player in the field of protein directed evolution^38,39^. The philosophy behind these works was trying to adopt high-throughput artificial DNA synthesis for the construction of semi-rational mutagenesis gene pools with wide coverage. Though great progress has been achieved in structure-guided mutagenesis and computer-aided directed evolution, useful mutations may still be dismissed at the beginning of these semi-rational approaches. Currently, solid-phase oligonucleotide synthesis by phosphoramidite chemistry is widely used in artificial DNA synthesis and is always carried out under strictly controlled conditions. However, base errors are still generated under specific conditions, such as high water content of DNA synthesis reagents, overexposure of deblocking reagents, and degradation of dNTPs (dG) phosphoramidites used in chain elongation reactions, etc^40–42^. Base errors generated during the chemical synthesis of oligonucleotides may be assembled into the synthesized DNA fragment and always need to be corrected^43^.

In this work, base errors generated during the chemical synthesis of oligonucleotides under specific conditions were treated as a source of random mutation, and an alternative approach of in vitro genetic diversification for directed evolution by error-prone Artificial DNA Synthesis (epADS) was suggested. Under test conditions, epADS has introduced diverse mutation types including base substitutions and indels randomly spanning the whole DNA sequence. Through this approach, 200~4000 folds of diversification in fluorescent strength have been achieved in genes encoding fluorescent proteins (EmGFP, Cherry, and mBanana). EpADS has also been successfully used to diversify regulatory genetic parts (*P*_*trc*_ promoter and tryptophan riboswitch), and synthetic gene circuits and even increase *Escherichia coli* DH5a’s tolerance to carbenicillin in a short period of time. In vitro generation of genetic diversity by epADS would be an alternative tool for directed evolution which may find useful applications in synthetic biology.

## Results

### Working principle of in vitro generation of genetic diversity for directed evolution by error-prone artificial DNA synthesis (epADS)

**Fig. 1:**
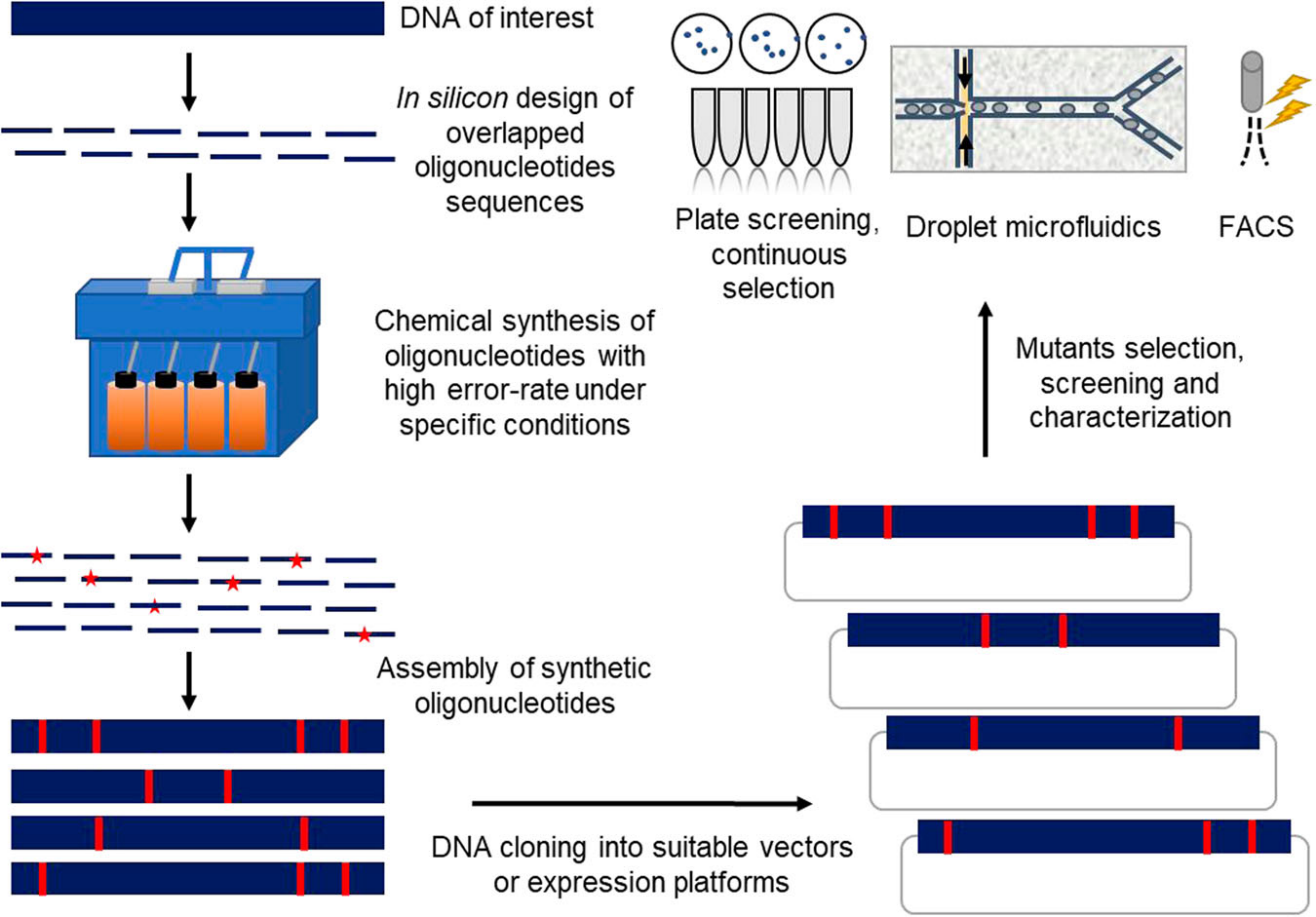
Working principle of in vitro generation of genetic diversity by error-prone artificial DNA synthesis (epADS). At the beginning of the in vitro generation of genetic diversity by epADS, the target DNA of interest, either it is a protein-coding gene, regulatory genetic parts like promoter and riboswitch, or synthetic circuit, was *in silicon* designed into overlapped oligonucleotides sequences covering the DNA of interest. And then, chemical synthesis of the designed oligonucleotides with a high error rate was carried out under specific conditions (like high water content of DNA synthesis reagents, mixed dNTPs monomers, and specific reaction programs tested in this work). During this process, base errors like indels, and base substitutions may take place in portions of the synthesized oligonucleotides. After that, the synthesized oligonucleotides were assembled into double-stranded DNA of interest by different methods like annealing (<200 bp) and PCR ( > 200 bp). For an even longer DNA sequence (>2 Kb), the target DNA sequence was broken into several blocks and each block was obtained via the above methods, then these blocks were integrated via fusion PCR or by the following cloning step. Thereafter, the obtained double-stranded DNA with possible errors incorporated was cloned into suitable vectors or expression platforms. Finally, the constructed library was ready for mutant selection, screening or characterization via different approaches like solid plate screening, continuous selection in liquid culture, droplet microfluidics screening or fluorescence-activated cell sorting (FACS).

The working principle of in vitro generation of genetic diversity for directed evolution by epADS has been depicted in Fig. 1. The whole process could be divided into five steps. At the first step of epADS, the DNA of interest was *in silicon* designed into overlapped oligonucleotides sequences covering the whole DNA sequence. The target DNA can be protein-coding genes, regulatory genetic parts like promoters and riboswitches, or synthetic gene circuits. In the second step, the designed oligonucleotides sequences were chemically synthesized under specific conditions (like high water content of DNA synthesis reagents, mixed dNTP monomers, and specific reaction programs tested in this work). During this process, base errors including indels and base substitutions may take place in some synthesized oligonucleotides. In the third step, the synthesized oligonucleotides were assembled into double-stranded DNA through different procedures like annealing (<200 bp) or PCR (>200 bp). For an even longer DNA sequence (>2 Kb), the target DNA sequence was broken into several blocks and each block was obtained via the above steps, then these blocks were integrated via fusion PCR or by the following cloning step. In the fourth step, the double-stranded DNA with possible errors incorporated was cloned into suitable vectors or expression platforms. Finally, the constructed library was ready for mutant selection, screening, or characterization via different approaches according to their specific needs.

**Fig. 2:**
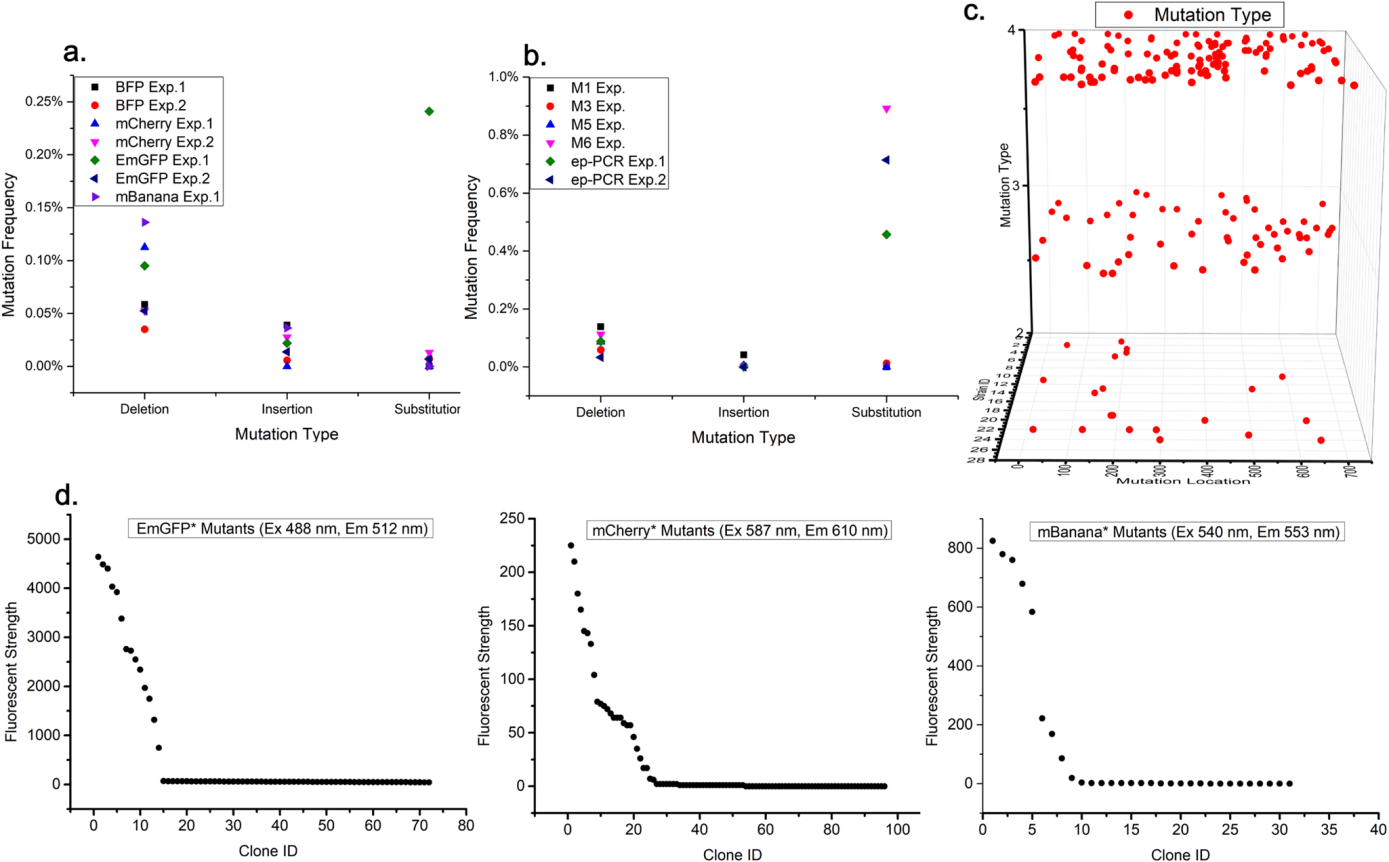
Characterization of mutations generated by epADS under different suboptimal conditions and phenotypic characterization of three genetic parts generated by epADS. **a** Types and frequency of mutations in four genes encoding fluorescent proteins (EmGFP, mCherry, BFP, and mBanana) generated by epADS with long-term used DNA synthesis solvents in the preliminary test. **b** Types and frequency of mutations in the gene encoding the fluorescent protein (EmGFP) generated by epADS in M1, M3, M5, and M6 test. A comparative test of in vitro generation of genetic diversity by ep-PCR was also carried out as described in “Methods” section. **c** Types and distributions of mutations in the gene encoding the fluorescent protein (EmGFP) generated by epADS of M6 test. Types of mutations were represented by numeric values on the Y-axis, 1: Base insertion (not observed); 2: Base deletion; 3: Base transition; 4: Base transversion. **d** Diversification in fluorescent strength of three genetic parts encoding fluorescent proteins EmGFP, Cherry, and mBanana modulated by epADS.

In the preliminary test, epADS of four genes encoding a fluorescent protein (EmGFP, mCherry, BFP, and mBanana) was carried out with long-term used DNA synthesis solvents. DNA sequences of these four genes were obtained from the GenBank database (https://www.ncbi.nlm.nih.gov/genbank/) and were listed in Supplementary Data 1. Overlapped oligonucleotides sequences covering each gene were designed with the online tool DNAWorks (https://hpcwebapps.cit.nih.gov/dnaworks/). The designed oligonucleotides sequences were also listed in Supplementary Data 1. Chemical synthesis of oligonucleotides was carried out as described in the “Methods” section with normal procedures except for the utilization of long-term used DNA synthesis solvents. The synthetic oligonucleotides were assembled into full-length genes by PCR and then cloned into the pEASY®-Blunt Zero Cloning vector. Sanger sequencing of plasmids extracted from transformed colonies was performed to identify possible mutations introduced by epADS. Two independent experiments were carried out for genes encoding EmGFP, Cherry, and BFP, while one independent experiment was successfully carried out for the gene encoding mBanana. About one hundred colonies have been sequenced for genes encoding BFP (103 colonies), mCherry (112 colonies), and EmGFP (99 colonies), while only 31 colonies have been successfully sequenced for the gene encoding mBanana. Results show that both indels and base substitutions were found in these genes produced by epADS (Fig. 2a). Most of the time, base deletion was the most frequent type of mutation, with an error rate ranging from 0.035% to 0.14% (Fig. 2a). Base insertion and substitutions were the second and third largest types of mutations (Fig. 2a). The total mutation frequency of these four genes (with a length of 0.8–1 Kb) was 0.05%–0.17%. The ratio of mutants among total sequenced colonies was all above 1/3, which indicated that epADS may be useful for in vitro generation of genetic diversity.

To facilitate the application of epADS for in vitro generation of genetic diversity, we gradually carried out four tests (M1, M3, M5, and M6) to characterize the mutation profiles of the gene encoding EmGFP generated by epADS under different conditions. In the M1 test, the time of the coupling reaction was reduced by 50% as compared with that of the standard synthesis reaction, and other parameters were unchanged. In M3 test, standard DNA synthesis solvent used in M1 test (newly opened) was replaced with a long-term used DNA synthesis solvent for epADS. In the M5 test, the “Hi drain” action in the washing step after the coupling reaction of the M3 test was deleted. In the M6 test, standard dNTPs monomers used in the M5 test were replaced with premixed dNTPs* reagents (including 99.0% w w^−^^1^ of the main dNTP component along with 0.33% w w^−^^1^ of each of the other three dNTP monomers, see “Methods” section). Mutations identified in these tests were listed in Table 1. Results showed that in M1, M3, and M5 tests, the ratio of mutants among sequenced colonies was 39.4%–60.0%, while the value of the M6 test was 100.0%. Results also showed that the most common type of mutation was deletions in M1, M3, and M5 tests (Fig. 2b). However, base substitution was the most common type of mutation in the M6 test, a higher mutation frequency (8.92 bp Kb^−^^1^) and random distribution of mutations has also been observed in this test (Fig. 2c). As a comparative study, in vitro generation of genetic diversity of the gene encoding mCherry by ep-PCR was also conducted to characterize its mutation profiles. Two independent ep-PCR tests with different amounts of template DNA (~50 ng plasmid DNA for Exp.1 and ~5 ng plasmid DNA for Exp.2) were carried out simultaneously. Mutations generated by ep-PCR were listed in Supplementary Data 2. A base substitution frequency of 0.71% was observed in ep-PCR Exp.2. This value was higher than that of ep-PCR Exp.1 (0.46%), but was lower than that of the M6 test. The total mutation frequency was also higher in ep-PCR Exp.2 as compared with ep-PCR Exp.1.

**Table 1:** Mutations generated during epADS of fluorescent protein-coding gene EmGFP under test conditions of M1, M3, M5, and M6

Batch of exp.	Sample ID	Mutation type	Mutant sequence ratio
M1	M1-2	1DelN70; 318InsT; 335DelTG; 442InsG; 698InsC	60.0%
	M1-3	180DelGAC; 192DelT	
	M1-4	180DelGAC; 192DelT	
	M1-5	570DelNx	
	M1-6	260DelN8	
	M1-9	483DelGT; 510DelC	
M3	M3-9	218DelN5	39.4%
	M3-11	193Del T; 553 DelCAG; 620DelC	
	M3-16	547InsC	
	M3-19	1 DelN77	
	M3-20	1DelN243	
	M3-22	396DelA; 411Del N9; A797C	
	M3-25	1DelN249; C634A	
	M3-26	328DelC	
	M3-29	1DelN243	
	M3-32	406DelA	
	M3-35	718DelN3	
	M3-38	C312A	
	M3-40	1 DelN77	
M5	M5-3; 19	72DelN4; 702DelN19	43.8%
	M5-4	223DelN16	
	M5-5	1DelN22	
	M5-6; 8; 10	226DelN2; 233DelA	
	M5-13	397DelG	
	M5-16	615DelG; 620DelC; 624DelN7	
	M5-25 ~ 28	19DelG	
	M5-38	31InsC	

Batch of exp.	Sample ID	Mutation type	Mutant sequence ratio
M6	M6-1	C41G ; A80T ; T161G ; 188DelC ; G229A ; G352C ; T377G ; A429T ; C558G ; A644T	100.0%
	M6-2	T33A; 49DelG; C188G; C255T; C443T; C589G; C611G	
	M6-3	204DelT; C269A; C498G; C504T; T587A	
	M6-4	204DelT; C269A; C498G; C504T; T587A	
	M6-5	C41T; A104T; 176DelC; A190G; T214A; A407C; A493T; T671G; T689C	
	M6-6	G331T; A421T; A449G; T453A; A542T; C651A; T674A	
	M6-7	C297T; C332G; A380T; A421C; G520A; T623A	
	M6-8	G32A; C170G; G383T; C412G; C438A; A449G	
	M6-9	G166A; C227T; G524T; T547A; G682C	
	M6-10	A71G; T93A; C180G; G466A; A488C; T496A; 590DelC; A669T	
	M6-11	10DelA; T89A; G129A; C309A; G383A; A428T; A512C; T582A; G631C	
	M6-12	T222G; A265T; C327G; A419T; G534C	
	M6-13	G100T; 157DelA; T319A; A364T; A434T; 513DelC; C532G; A545G; T656C; C692T	
	M6-14	G24C; C116A; 139DelT; A287T; G404C; C420G; T587C; T684C	
	M6-15	C367T; G383C; G555A; C612T; C678T; T681A	
	M6-16	G228A; A423T; C447T; A512G; C612T; T627C; T681A	
	M6-17	G32A; C170G; G383T; C412G; C438A; A449G	
	M6-18	C297T; C332G; A380T; A421C; G520A; T623A	
	M6-19	G129T; 184DelG; 188DelC; T199G; A556G; T621A	
	M6-20	A130T; A200T; A322C; T333A; A364T; A394C; 398DelA; A434C; A519T; A610C; G624A; 628DelA	
	M6-21	G229A; G275C; T333G; T384A; A487G; C594G	
	M6-22	14DelG; C29T; 124DelA; 229DelG; 289DelC; C309G; A396C; G397T; C562T	
	M6-23	G49T; A99T; T114G; C153G; A178T; A209G; T254G; C435A; A478G; 494DelACT	
	M6-24	T143C; A238T; C269A; C283G; 298DelT; C328T; T625G; 652DelC; C672G	
	M6-25	C45A; A159C; G165T; A364C; A389G; A499G	
	M6-26	C141G; C181T; A200G; G564T; C692G	

Note: M1: dNTPs (Hongen Biotech) and fresh DNA synthesis solvents (Hebei DNAchem Biotech) were used in the oligonucleotides synthesis with a 50% reduction in coupling time of standard synthesis program on a Dr. oligo192 platform; M3: dNTPs (Hongen Biotech) and stored DNA synthesis solvents (Hebei DNAchem Biotech) were used in the oligonucleotides synthesis with a 50% reduction in coupling time of standard synthesis program on a Dr. oligo192 platform; M5: dNTPs (Hongen Biotech or Sigma Aldrich) and stored DNA synthesis solvents (Hebei DNAchem Biotech) were used in the oligonucleotides synthesis with a 50% reduction in coupling time of standard synthesis program and deletion of the “Hi drain” step after coupling reaction on a Dr. oligo192 platform; M6: premixed dNTPs (Hongen Biotech or Sigma Aldrich) and stored DNA synthesis solvents (Hebei DNAchem Biotech) were used in the oligonucleotides synthesis with a 50% reduction in coupling time of standard synthesis program and deletion of the “Hi drain” step after coupling reaction on a Dr. oligo192 platform.

### In vitro generation of genetic diversity of genetic parts and synthetic gene circuits by epADS

To demonstrate the feasibility of in vitro generation of genetic diversity for directed evolution by epADS, we characterized the fluorescent profile of transformed colonies of genes encoding EmGFP, Cherry, and mBanana obtained through epADS in the preliminary test. About one hundred colonies from genes encoding EmGFP (72 colonies), mCherry (96 colonies) and 31 colonies from the gene encoding mBanana were analyzed; data were presented in Fig. 2d. A range of 0–4640 in fluorescent strength was observed in colonies of gene encoding EmGFP (Ex 488 nm, Em 512 nm). For mCherry, a range of 0–225 in fluorescent strength has been detected (Ex 587 nm, Em 610 nm). As for mBanana, the range of fluorescent strength observed was 0–825 (Ex 540 nm, Em 553 nm).

**Fig. 3:**
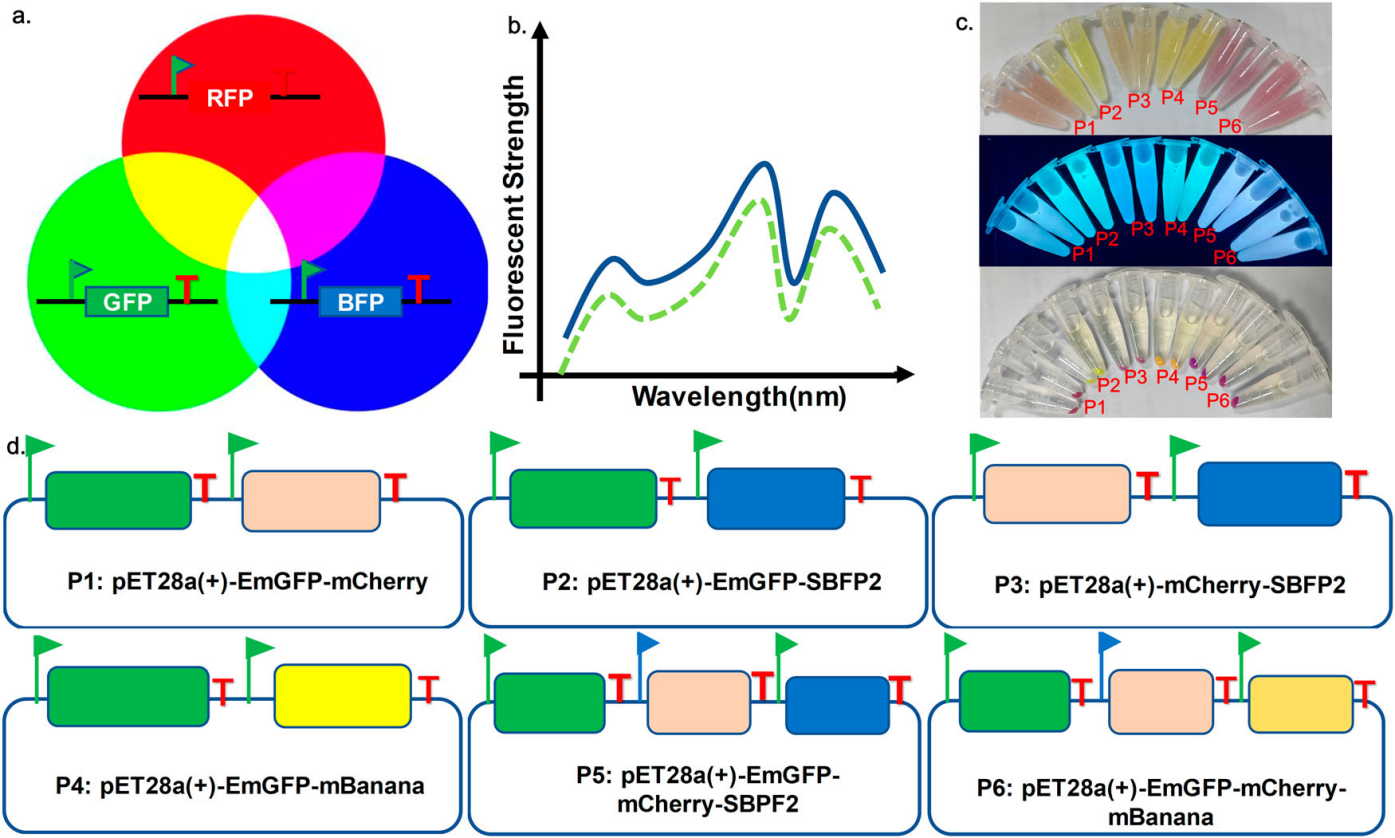
Construction of serials of synthetic gene circuits encoding a red-green-blue (RGB) bio-palette system. **a** Working principle of the Red-Green-Blue (RGB) bio-palette system. **b** Schematic illustration of the fluorescent phenotype of the RGB bio-palette system. **c** Color of cultures and cell pellets of serials of synthetic gene circuits encoding the RGB bio-palette system under illumination with room light or ultraviolet light. **d** Structure design of serials of synthetic gene circuits (P1~P6) encoding the RGB bio-palette system, otherwise indicated, *T7* promoter, *P*_*trc*_ promoter, and *T7* terminator was used for regulation of the expression of specific genetic parts.

**Fig. 4:**
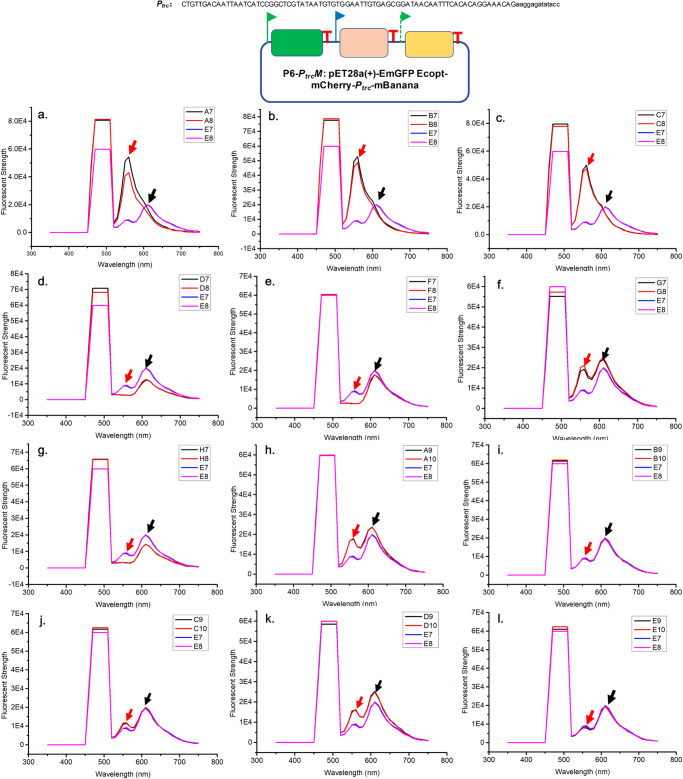
Phenotype characterization of regulatory genetic parts (*P*_*trc*_ promoter) modulated by epADS. Fluorescent profiles of 12 colonies (**a**–**i**) from the P6-*P*_*trc*_*M* mutant library generated by epADS of *P*_*trc*_ promoter were monitored to reflect its regulation strength on the downstream gene (mBanana). Fluorescent spectrum analysis was carried out with an excitation wavelength of 488 nm. The peak at 553 nm represented the emission signal of mBanana (red arrow), and the peak at 610 nm represented the emission signal of mCherry (black arrow).

**Fig. 5:**
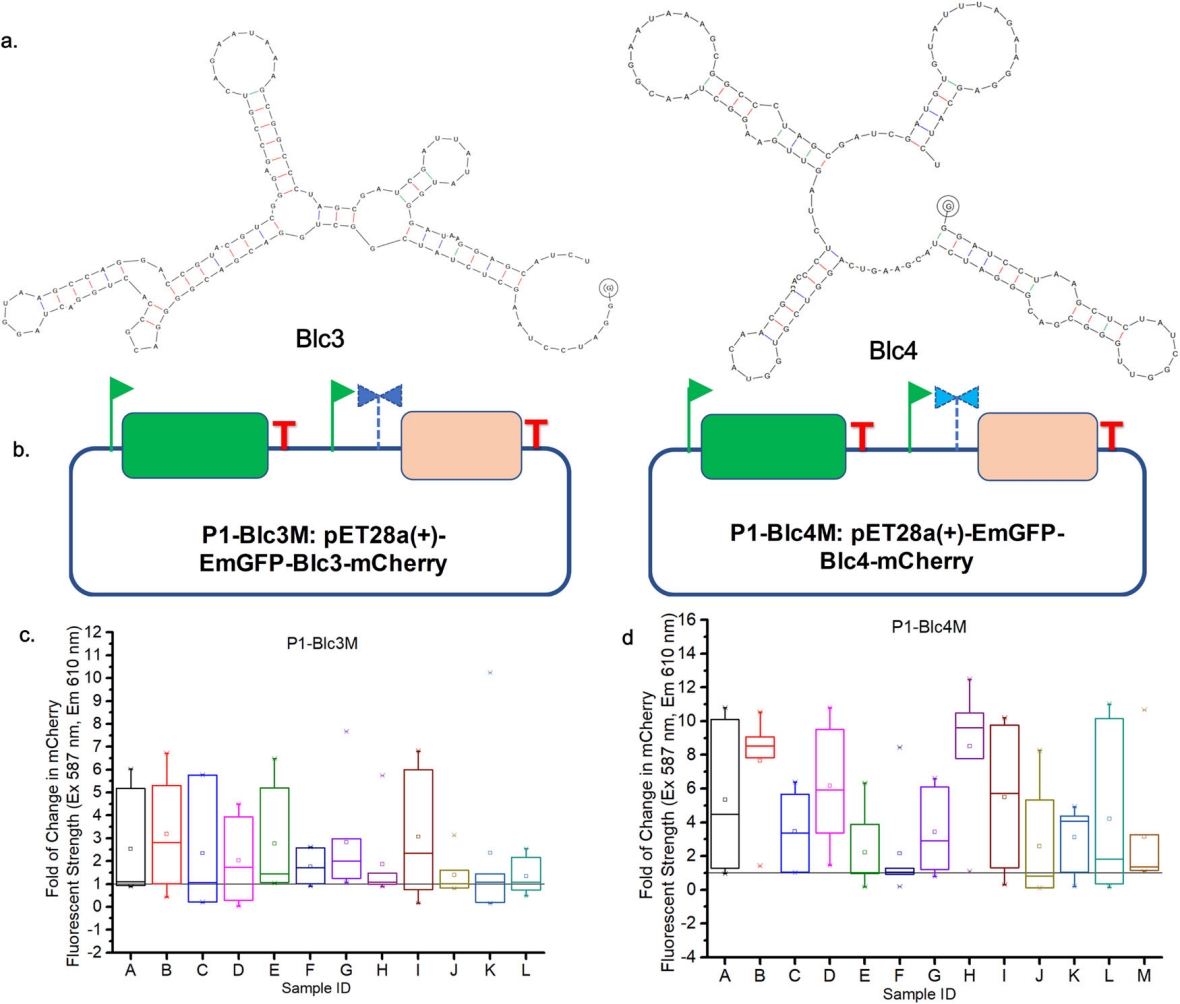
Phenotype characterization of regulatory genetic parts (tryptophan riboswitches) modulated by epADS. **a** Structure illustration of two tryptophan riboswitches Blc3 and Blc4. **b** Structure design of two synthetic gene circuits P1-Blc3M and P1-Blc4M used to evaluate modulation of tryptophan riboswitches by epADS. **c**, **d** Fold of change in fluorescent strength of mCherry regulated by tryptophan riboswitch with/without tryptophan addition was determined to reflect its regulation activity on the downstream gene. 12 colonies from the P1-Blc3M mutant library and 13 colonies from the P1-Blc4M mutant library cultured in M9 medium with/without tryptophan (1 mM) were assayed for their fluorescent strength.

To further our understanding of the application potential of epADS in synthetic biology, we constructed serials of synthetic gene circuits encoding a Red-Green-Blue (RGB) bio-palette system (Fig. 3a, b). The RGB bio-palette system was constructed based on a pET28a (+) expression vector and various combinations of genetic parts encoding fluorescent proteins (EmGFP, mCherry, BFP (SBFP2) or mBanana) under the control of *T7*, *P*_*trc*_ promoter or other regulatory genetic parts. Briefly, four two-gene synthetic gene circuits (P1~P4) and two three-gene synthetic gene circuits (P5, P6) were constructed (Fig. 3d). Color of liquid cultures under different illumination conditions (normal room light and UV lamp with a wavelength of 254 nm) and cell pellets of the RGB bio-palette system transformed into host cells were presented in Fig. 3c. We also characterized the fluorescent profile of the RGB bio-palette system, data was presented in Supplementary Files Fig. 1–5.

Based on the constructed RGB bio-palette system, the promoter of mBanana (*P*_*trc*_) in P6 was selected for epADS. The 87 bp *P*_*trc*_ promotor and its flanking sequence (~30 bp) were *in silicon* designed into four overlapped oligonucleotides sequences (P5-R2: ATGCTCGGCCTACTAATTAACAGTTGTCCATCGCCAGTGCGACGCGCATTGGT

GGTGTG; mCherry block-P1: CTGTTGACAATTAATCATCCGGCTCGTATAATGTGTGGAATTGTGAGCGGATAA

CA; mCherry block-P2: ggtatatctccttCTGTTTCCTGTGTGAAATTGTTATCCGCTCACAATTCCACACATTA; mBanana block-P3: ATTTCACACAGGAAACAGaaggagatataccATGGTGAGCAAGGGCGAGGAGAATAACA) and produced by epADS as those described in M5 test. PCR assembly was carried out to obtain the insert fragments for integrating into the P6 synthetic gene circuit. Types of mutations were identified by Sanger sequencing of plasmids extracted from transformants of the P6 synthetic gene circuit (P6-*P*_*trc*_*M*). In three independent experiments, 5, 10, and 15 colonies were sequenced and both deletions and insertion were discovered in *P*_*trc*_ promoter and its flanked sequence of P6-*P*_*trc*_M. The ratio of mutant sequences was 6.7%–40.0% in these three independent experiments (Table 2). The fluorescent profile of colonies from the P6-*P*_*trc*_M mutant library was also determined. To avoid intensive signal interference from the excitation wave, the excitation of test cultures was carried out at 488 nm. The fluorescent strength of emission at 553 nm (a secondary emission peak of P6-*P*_*trc*_M when excited at 488 nm) was monitored to reflect the expression level of mBanana regulated by the *P*_*trc*_ promoter. In general, both increase and decrease in the fluorescent strength of mBanana have been observed in P6-*P*_*trc*_*M* produced by epADS. Among the 12 tested mutants, 7 mutants have shown various degrees of increased fluorescent strength and the highest level was 5.42 folds of the wild-type control (Fig. 4a–c, f, h, k, j). Three mutants have shown decreased fluorescent strength as compared with wild-type control (Fig. 4d, e, g). There were also two mutants that didn’t show any significant changes in fluorescent strength as compared with wild-type strain (Fig. 4i, l). In general, a range of 0.28–5.42 folds of changes in regulation strength of *P*_*trc*_ promoters derived from epADS has been observed. On the contrary, the regulation strength of another wild-type *P*_*trc*_ promoter in the same plasmid has shown a relatively narrow range of divergence (0.64–1.26 folds of changes).

**Table 2:** Types of errors generated in epADS of *P*_*trc*_ promoter

Sample ID	Mutation type	Mutant sequence ratio
P6M1	mBanana CDS 21DelG	20.0%
P6M2	48DelC; mBanana CDS 18DelG	6.7%
P6M3	45DelG	40.0%
P6M7	37DelG; mBanana CDS 15InsG	
P6M8	26DelG	
P6M12	47InsG

We also tested the application of epADS for in vitro genetic diversification of other regulatory genetic parts. Another two synthetic gene circuits (P1-Blc3M, P1-Blc4M) were constructed based on P1 plasmid from the RGB bio-palette system and two tryptophan riboswitches^44,45^ (Blc3 and Blc4) produced by epADS (Fig. 5). DNA sequences of Blc3 and Blc4 used for epADS have been listed in Supplementary Data 1. Two long oligonucleotides have been designed for epADS of Blc3 (pG-mC block3-PF: gggatcctaagctctatcggCTGGACGACGGGGACGCCACTGGACTAGGTAAGCCAGGACCGTACGTCGGGAGCCGTCAGAATAaagcggccctagcgatcgatTATATGGGATaaggagcatct; pG-mC block3-PR: agatgctccttATCCCATATAatcgatcgctagggccgcttTATTCTGACGGCTCCCGACGTACGGTCCTGGCTTACCTAGTCCAGTGGCGTCCCCGTCGTCCAGccgatagagcttaggatccc) and Blc4 (pG-mC block4-PF: gggatcctaagctctatcggTTGGGCGACGGGATCTACGAAGTCAGGTCGTGGTACAACGAAACCCTCTAGTTGAAGGCTAACGGAATAaagcggccctagcgatcgatGTGTATTTAGaaggagcatct; pG-mC block4-RF: agatgctccttCTAAATACACatcgatcgctagggccgcttTATTCCGTTAGCCTTCAACTAGAGGGTTTCGTTGTACCACGACCTGACTTCGTAGATCCCGTCGCCCAAccgatagagcttaggatccc) respectively. The synthetic oligonucleotides pairs were produced by epADS according to the M5 test and annealed into double-stranded DNA and integrated into the P1 plasmid via seamless assembly. Mutations in P1-Blc3M and P1-Blc4M were identified by Sanger sequencing of plasmids extracted from transformants of P1-Blc3M (168 colonies) and P1-Blc4M (129 colonies). Results showed that base substitutions have also been detected along with deletions, and the ratio of mutant sequences was 16.7% and 19.4% respectively (Table 3). Then we assayed their responses to tryptophan addition by monitoring the fluorescent strength of their regulated genes (mCherry). 12 strains of P1-Blc3M and 13 strains of P1-Blc4M with mutated riboswitches generated by epADS were assayed for their fluorescent strength with or without the supplement of 1 mM tryptophan during cultivation. About 50% of mutated Blc3 riboswitches have shown nearly 5 folds of increase in median fluorescent strength after tryptophan addition (Fig. 5c: A–E, I). Three mutants have shown less than 3 folds of increase in median fluorescent strength after tryptophan addition (Fig. 5c: F, G, L). There was also one mutant have showed a little decrease in median fluorescent strength after tryptophan addition, and the other two mutants had no obvious changes in this value. As for Blc4, about 50% of mutated riboswitches have shown 8 to 10 folds of increase in median fluorescent strength after tryptophan addition (Fig. 5d: A, B, D, H, I, L). Another 50% of mutated Blc4 riboswitches (Fig. 5d: E, G, J, K, M) have shown 2–6 folds of increase in median fluorescent strength after tryptophan addition, and only one mutant didn’t show any change in this value.

**Table 3:** Types of errors generated in epADS of tryptophan riboswitches Blc3 and Blc4

Sample ID	Mutation type	Mutant sequence ratio
P3M82	122DelA	16.7%
P3M28	13DelT	
P3M4, P3M24, P3M50, P3M73, P3M103; P3M107, P3M137	16DelA	
P3M3, P3M62, P3M161	36DelGCC	
P3M122	38DelC	
P3M80	46DelT	
P3M42, P3M133, P3M174	5DelT	
P3M26	65DelCGT	
P3M143	A103G	
P3M68	C101A	
P3M6, P3M18, P3M30, P3M59, P3M86, P3M112	Large del	
P3M67	mCherry CDS 16DelC	
P3M182	mCherry CDS 20InsG	
P4M24	11DelG	19.4%
P4M52	131DelA	
P4M38, P4M85	27DelG	
P4M133	53DelG	
P4M9	57DelAT	
P4M52	66DelC	
P4M20, P4M122	76DelA	
P4M6, P4M82	8DelT	
P4M38, P4M85	8InsC	
P4M26, 28, P4M130	A75G	
P4M32	C59A	
P4M43	G30T	
P4M30	G85A	
P4M106	Large Del	
P4M11, P4M120	mCherry CDS 12DelG	
P4M39	mCherry CDS 20DelA	
P4M61	mCherry CDS -3DelN13	
P4M52	mCherry CDS 6DelG	

**Fig. 6:**
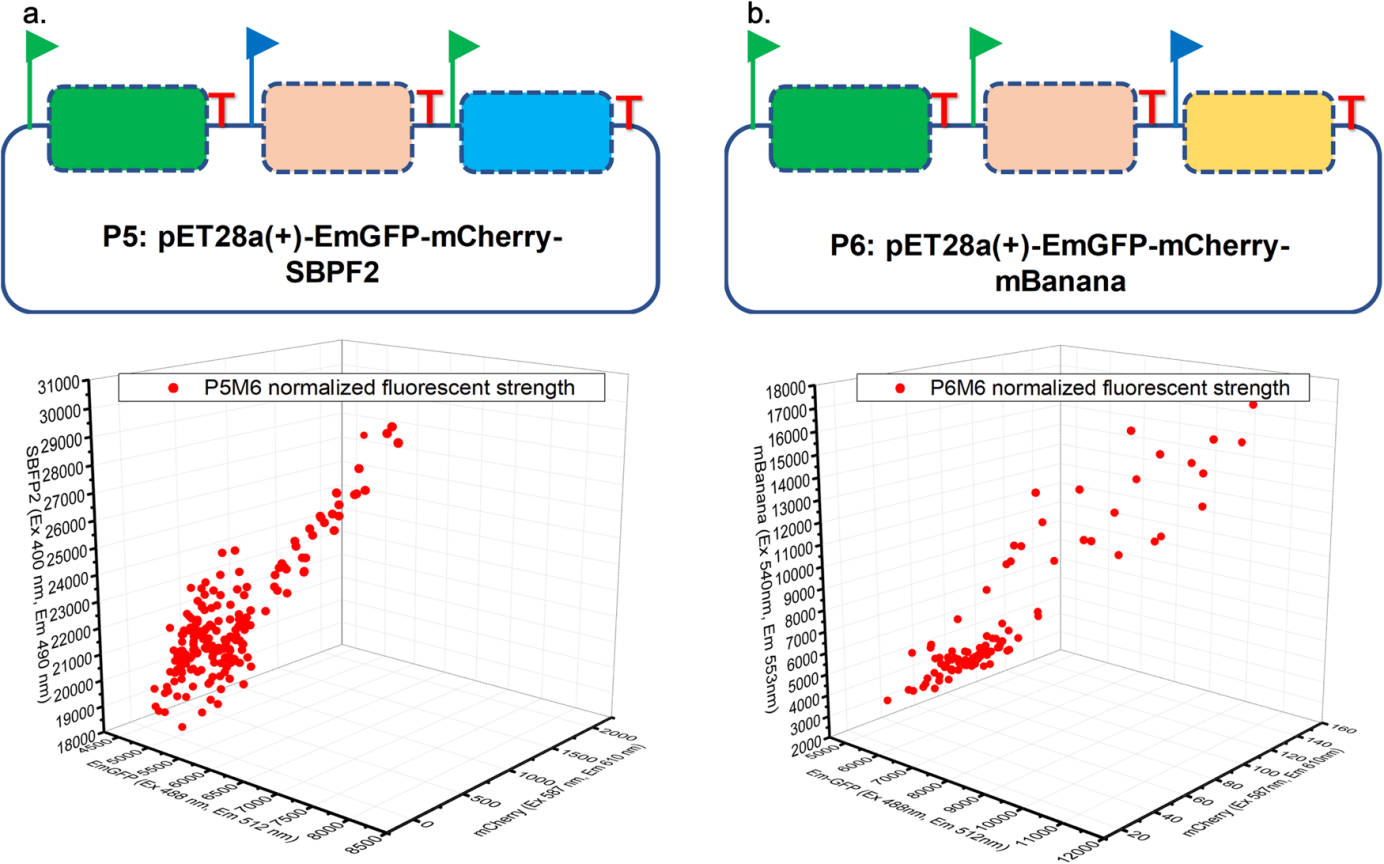
Phenotypic characterizations of two synthetic gene circuits encoding an RGB bio-palette system modulated by epADS. **a** Synthetic gene circuit P5 was constructed based on the pET28a(+) plasmid with the incorporation of genes encoding EmGFP, Cherry, and SBFP2 regulated by the *T7* promoter and *P*_*trc*_ promoter respectively. The fluorescent strength of EmGFP, Cherry, and SBFP2 from colonies of the P5M6 mutant library generated by epADS was determined. **b** Synthetic gene circuit P6 was also constructed based on the pET28a(+) plasmid with the incorporation of genes encoding EmGFP, Cherry, and mBanana regulated by *T7* promoter and *P*_*trc*_ promoter respectively. The fluorescent strength of EmGFP, Cherry, and SBFP2 from colonies of the P6M6 mutant library generated by epADS were determined.

Besides the abovementioned genetic parts, we further carried out epADS of two multi-gene synthetic gene circuits (P5 and P6) from the RGB bio-palette system to modulate their phenotypes. The mutant libraries were constructed by an iterative replacement of wild-type genes encoding EmGFP, Cherry, and SBFP2 or mBanana in P5 and P6 plasmids with their alternatives produced by epADS as described in “Methods” section. These alternative genes encoding EmGFP, Cherry, and SBFP2 or mBanana were produced by epADS according to M6 test conditions. The fluorescent strength of EmGFP, Cherry, and SBFP2 or mBanana of 192 colonies from the finally constructed mutant library (P5M6-Run3 and P6M6-Run3) were determined and plotted in a 3D-scatter graph. Results showed that a scattered distribution of plotted dots in the 3D graph was observed, which reflected a wide range of phenotypic diversification in these synthetic gene circuits derived from epADS (Fig. 6). For the P5M6-Run3 library, a range of 0~2000 in fluorescent strength was observed for mCherry, while only about 2 folds of variance in fluorescent strength of EmGFP or SBFP2 was detected (Fig. 6a). For the P6M6-Run3 library, a range of 0~160 in fluorescent strength was observed for mCherry, and about 2 to 9 folds of variance have been observed in fluorescent strength of EmGFP and mBanana respectively (Fig. 6b).

### Increasing tolerance of *Escherichia coli* to carbenicillin by epADS of *bla* gene encoding beta-lactamase and adaptive laboratory evolution (ALE)

**Fig. 7:**
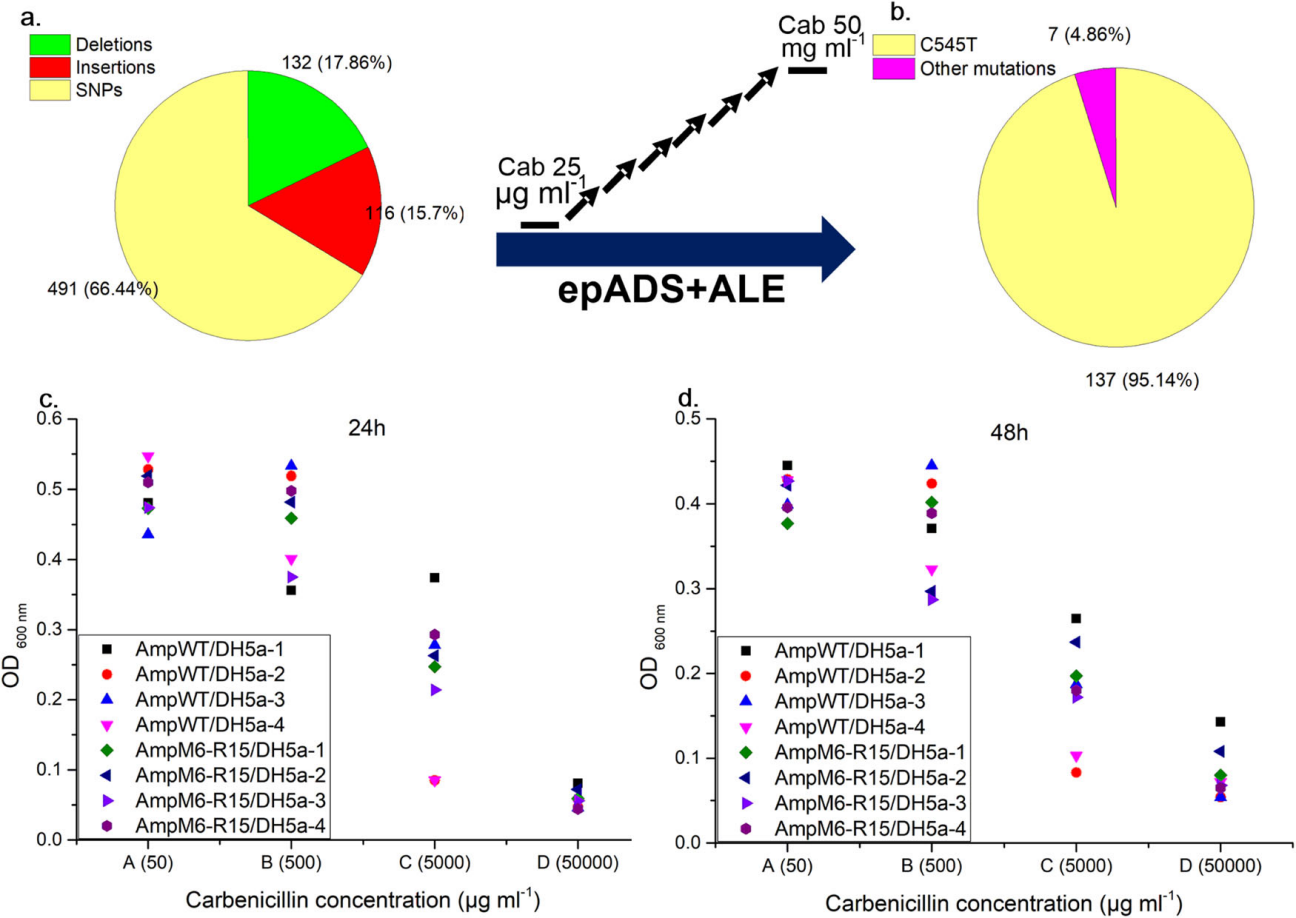
Combination of epADS with ALE for increasing microbial tolerance to antibiotics. The *bla* gene generated by epADS was cloned and transformed into *E. coli* DH5a cells to obtain the parent culture for ALE with a stepwise increase of carbenicillin concentration. **a** Distribution ratio of different types of mutations in the starting library of ALE (AmpM6-R0). **b** Distribution ratio of different types of mutations in the evolved library of ALE (AmpM6-R15). Growth of cultures with the evolved *bla* gene after 24 h (**c**) and 48 h (**d**) of cultivation under various concentrations of carbenicillin. Results presented were obtained from tests with three biological replicates of the ALE experiment and four replicates of the growth inhibition assay.

Here we also carried out a combined application of epADS with ALE to test its potential of modulating microbial phenotypes such as improving host tolerance to antibiotics (carbenicillin). *Bla* gene encoding beta-lactamase was selected for epADS according to M6 test conditions. The DNA sequence of the *bla* gene and the *in silicon* designed oligonucleotides sequences for epADS were all listed in Supplementary Data 1. The *bla* gene fragment produced by epADS was cloned into a modified p15C vector (P15C-M1-37-Kan) as described in “Methods” section. The *bla* gene mutant library generated through epADS was not screened on LB plates immediately after its construction but was handed out for ALE as those described in “Methods” section. During the 2 weeks ALE process with a stepwise increase of carbenicillin concentration from 25 µg ml^−^^1^ to 50 mg ml^−^^1^, two of the three parallel evolved cultures lost growth ability at the fifth round of re-transfer. The sixth round of cultivation was continued with only one growth culture with three replicates. After the ALE process, three tubes of evolved cultures were sprayed and streaked on LB agar plates with 5 mg ml^−^^1^ and 10 mg ml^−^^1^ of carbenicillin (along with 25 µg ml^−^^1^ of kanamycin) separately. Colonies growth on these LB agar plates were picked and recheck for growth in 48-well plates as those described in “Methods” section. 50 µl of culture from the top 30 colonies in cell density were re-inoculated into an LB medium containing 50 mg ml^−^^1^ of carbenicillin to evaluate its growth potential under high carbenicillin concentration. Obvious growth (OD _600 nm_ 0.5~1.0) was observed in four colonies (AmpM6-R15-19, 20, 22, and 30) after 43 h of cultivation, and no growth was observed in the wild-type strain. The mutation profile of the starting mutant library of the *bla* gene generated by epADS (AmpM6-R0) was also characterized by Sanger sequencing. Plasmids extracted from 61 colonies of the starting library were sequenced and 91.8% of them were mutants. Among these mutants, a higher ratio (66%) of base substitutions was observed as compared with base deletions (18%) and insertions (16%) (Fig. 7a). A range of 1–36 point mutations was observed in mutants of the starting library (Supplementary Data 3). Plasmids extracted from 159 colonies of the evolved cultures were also sequenced and the most prominent mutation was C545T base substitution (Fig. 7b). Detailed list of mutations has been provided in Supplementary Data 4. Plasmids from those four cultures with the highest tolerance to carbenicillin were also extracted for Sanger sequencing, and C545T base substitution was also found in all those colonies. This mutation has led to an amino acid substitution of A182V in the beta-lactamase protein sequence which may finally lead to an increase in carbenicillin tolerance. These plasmids with C545T base substitution were retransformed into wide-type *E. coli* DH5a competent cells, an average of 23.7% of increase was observed in the growth of transformed colonies (AmpM6-R15/DH5a) as compared with wild-type control (AmpWT/DH5a) after 24 h of cultivation in LB media containing 5000 µg ml^−^^1^ of carbenicillin (Fig. 7c). This advantage was also lasted to 48 h of cultivation (Fig. 7d).

## Discussion

Artificial DNA synthesis is a foundational technology in synthetic biology which is based on the assembly of synthetic oligonucleotides into double-stranded DNA. Errors generated during synthetic oligonucleotides production and the assembly process could be incorporated into the final DNA of interest. These incorporated errors may provide a source of mutation for in vitro genetic diversification of the synthetic DNA. Previously, assembly of designed oligonucleotides (ADO) has already been developed for gene recombination of target genes for directed evolution^46^. Generation of base errors during chemical synthesis of oligonucleotides under suboptimal conditions has also been reported^40,41,47,48^. In this work, an alternative method entitled error-prone Artificial DNA Synthesis (epADS) was developed for in vitro generation of genetic diversity of genetic parts and synthetic gene circuits.

The mutation profile of sample DNA generated by epADS under different conditions was investigated. In the preliminary test of epADS with four fluorescent protein-coding genes, the average error rate (1 ~ 2 bp per Kb) was a litter higher than the average mutation rate of regular ep-PCR reactions (0.1~1 bp per Kb)^49^. The ratio of mutant sequences was all above 1/3 which has indicated the feasibility of epADS for the screening of interested phenotypes. In M1 ~ M6 tests, when epADS was carried out under suboptimal reaction conditions such as the use of long-term used DNA synthesis solvents (Acetonitrile, with a water content of 20 ~ 900 ppm), shortened coupling reaction time or withdraw of the “Hi drain” washing step after coupling reaction and their combinations, base deletions were the most common type of mutation. These results were consistent with findings of previous work^40^. In M1, M3, and M5 tests, base insertions and substitutions were less frequently occurred as compared with deletions. However, when low purity dNTP monomers (like dNTPs mixtures premixed with other dNTP monomers) were used in epADS, the frequency of base substitutions increased significantly (8.92 bp per Kb in the M6 test). This value is even higher than the comparative ep-PCR test (7.1 bp per Kb). Base substitutions may finally lead to amino acid changes in translated protein sequence and is very important for the success of protein directed evolution. This result indicated that the M6 test may have provided suitable conditions for epADS of those protein-coding genes. Results from the M1~M6 test have also demonstrated that different mutation profiles can be generated by epADS carried out under different suboptimal conditions. Thus, epADS could be carried out under specific conditions to reach the most effective outcome for the DNA of interest. As cost is also a factor of consideration when choosing suitable methods for the construction of mutant libraries, the cost of epADS was comparable or even lower than that of ep-PCR as no pre-construction and sequencing confirmation of template DNA was required. The major cost of epADS lies on the synthetic oligonucleotide production which has decreased to about less than 0.05 $ per base. Construction of a mutant library of a 1 Kb DNA fragment by in vitro generation of genetic diversity through epADS may cost about 100$ which is acceptable for most laboratories.

We tested the application of epADS for in vitro genetic diversification of genetic parts and synthetic gene circuits. Results from the abovementioned preliminary test have shown the mutation profiles of fluorescent protein-coding genes, which has been predominated by base deletions along with a lower ratio of base insertions and substitutions. Phenotype analysis by monitoring the fluorescent strength of these colonies has shown a wide range of coverage (3 orders in value, from 1 to ~5000). This result is reasonable as many deleterious mutants may be generated by base deletions, and their effects on the fluorescent strength may also be different. The range of diversity in fluorescent strength of mCherry and mBanana generated by epADS was lower as compared with that of EmGFP. This phenomenon may be caused by the intrinsic high level of fluorescent activity of EmGFP protein, and may also correlate to the lower mutation rate in DNA sequence encoding mCherry and mBanana. For genetic diversification of protein-coding genes, base substitutions are usually the most effective mutations. Thus, epADS of these types of genetic parts could be carried out as those described in the M6 test.

In vitro generation of genetic diversity of synthetic gene circuits can be carried out by epADS of the coding sequence (CDS) region, and/or the regulatory genetic parts. For the CDS region, similar procedures and reaction conditions for epADS of protein-coding genes could be used. Iterative gene replacement with DNA fragments generated by epADS could also be used for multiple CDS modifications in synthetic gene circuits. As reported in this work, in vitro genetic diversification of P5 and P6 by epADS of three fluorescent protein-coding genes has shown a wide range of diversification in the fluorescent phenotype which may modify the general behavior of the synthetic gene circuit. epADS of regulatory genetic parts could be carried out under other suboptimal conditions to obtain the best result, like high water content of DNA synthesis solvents, less tight control of the moisture content of the working environment, or even suboptimal reaction programs. Under these conditions, deletions or insertions would be the most prominent type of errors generated by epADS. As we already know, besides the highly conserved regions in regulatory genetic parts like promoters, riboswitches, and other *cis-*acting elements, the distance between these conserved elements and their regulated genes is also important for their function. Indels introduced by epADS under the abovementioned conditions could enrich the mutation types and screening space of those regulatory genetic parts. In this work, in vitro generation of genetic diversity of the regulatory genetic parts (including *P*_*trc*_ promoter and tryptophan riboswitches) by epADS was carried out according to M5 test conditions. Under test conditions, the most frequent errors introduced into *P*_*trc*_ promoter and tryptophan riboswitches were base deletions, while base substitutions have also been observed in tryptophan riboswitches. Phenotype assay has also revealed the effect of in vitro genetic diversification of tested promoters and riboswitches by epADS. Phenotype diversification was more obvious in the tryptophan riboswitches (2~10 folds of increase in regulation strength) generated by epADS as compared with the *P*_*trc*_ promoter (0.28~5.42 folds of changes in regulation strength). This may be related to the shorter length of promoter DNA sequence and low mutation rate under test conditions. The longer DNA sequence of riboswitches may have a bigger mutational space which finally also leads to more diversified phenotypes. When epADS was carried out under other suboptimal conditions with higher error rates and larger mutant pool sizes, a better coverage of promoter strength may also be achieved. EpADS of those regulatory genetic parts could be implemented independently or in combination with other genetic parts in plasmids, synthetic gene circuits, or even whole genomes to further explore their phenotype potential.

Generation of genetic diversity is the critical step of directed evolution which has been widely used for the engineering of proteins, enzymes, metabolic pathways, and synthetic gene circuits. In the above text, epADS has shown its effectiveness of in vitro generation of genetic diversity of different types of genetic parts and synthetic circuits. EpADS of protein-coding genes can introduce all types of errors randomly throughout the whole sequence, such as base deletions, insertions, and substitutions. Theoretically, bias may not exist or could be prevented by using proper dNTPs mixtures. Besides common base substitutions, epADS can also introduce Indels conveniently, which may be important for the engineering of regulatory genetic parts or some special proteins (like antibodies). The ratio of mutated sequence can reach a relatively high level (like as high as 100%) which is convenient for downstream screening. In an additional test, epADS has also been successfully used along with ALE for rapid adaptive evolution of *E. coli* tolerant to high concentrations of carbenicillin by in vitro genetic diversification of a plasmid-borne *bla* gene encoding beta-lactamase. Compared with the classic ALE process started with a single parent strain, the genetically diversified starting culture may accelerate the adaptive evolution process. EpADS can also be used in combination with semi-rational mutagenesis to enhance the diversity of those smart libraries. Like other genetic diversification methods, epADS may also be implemented in combination with other in vitro (like ep-PCR and DNA shuffling) or in vivo evolution methods (like ALE) to further expand its application in synthetic biology. One critical step of epADS is the error-prone synthesis of oligonucleotides with an oligonucleotides (DNA) synthesizer under specific conditions. In the past decade, important progress has been reported in the investigations of high-throughput, miniatured, open-source, and personalized oligonucleotides (DNA) synthesizers which make them accessible to more regular bio-labs^50–52^. Thus, epADS could also be implemented in more regular bio-labs besides those DNA synthesis centers.

In summary, an alternative method of in vitro generation of genetic diversity for directed evolution by error-prone artificial DNA synthesis (epADS) was developed to modulate various genetic parts, synthetic gene circuits, and even microbial cells. It has shown the merits of rich mutation types, near-random distribution, low level of parent sequence contamination, and ease of execution. In vitro generation of genetic diversity of various genetic parts, synthetic circuits, and microbial cells by epADS have shown various levels of phenotypic modification which may have fruitful applications in synthetic biology and metabolic engineering.

## Methods

### Materials and medium

Otherwise indicated, LB media (10 g L^−^^1^ tryptone; 5 g L^−^^1^ yeast extract; 10 g L^−^^1^ NaCl) and agar plate (LB media supplemented with 1.5% w v^−^^1^ agar powder) were used for bacterial cultivation and phenotype characterization. SOC media (20 g L^−^^1^ tryptone; 5 g L^−^^1^ yeast extract; 0.5 g L^−^^1^ NaCl; 0.186 g L^−^^1^ KCl; 0.952 g L^−^^1^ MgCl_2_; 3.6 g L^−^^1^ glucose) was used in transformation experiment for cell suspension when constructing the multi-gene synthetic circuits or gene libraries. M9 minimal salt media (6.78 g L^−^^1^ Na_2_HPO_4_; 3 g L^−^^1^ KH_2_PO_4_; 0.5 g L^−^^1^ NaCl; 1 g L^−^^1^ NH_4_Cl; 4 g L^−^^1^ glucose; 0.24 g L^−^^1^ MgSO_4_; 0.011 g L^−^^1^ CaCl_2_; 0.1% v v ^−^^1^ trace element solution) was used for phenotypic characterization of tryptophan riboswitches generated by epADS^53,54^. Tryptone and yeast extract were purchased from Oxoid Limited (UK), NaCl and kanamycin sulfate were purchased from Sangon Biotech (Shanghai, China), and carbenicillin disodium salt was purchased from Beijing Solarbio Co., Ltd. (Beijing, China). Molecular biology reagents like Q5® DNA polymerase, DpnI restriction enzyme, and T4 DNA ligase were purchased from NEB Corporation (USA). The QuickMutation™ kit used for ep-PCR was purchased from Beyotime Biotechnology (Shanghai, China). pEASY®-T5 Zero Cloning Kit, pEASY®-Blunt Zero Cloning Kit, and competent cells were obtained from TransGen Biotech Co., Ltd. (Beijing, China), seamless DNA cloning kit was obtained from Biomed Corporation (Beijing, China). Spin Column DNA Gel Extraction Kit and Plasmid DNA Extraction Kit were purchased from Sangon Biotech (Shanghai, China). Normal oligo DNA synthesis and sequencing services were commercially provided by Beijing Tsingke Biotech Co., Ltd. (Beijing, China).

### Procedures and reaction conditions tested for epADS and the comparative test of in vitro generation of genetic diversity by ep-PCR

Procedures of epADS were derived from normal artificial gene synthesis. In the beginning, the DNA sequence for in vitro genetic diversification was obtained from bioinformatics database and *in silicon* designed into overlapped oligonucleotides sequences with online tools (such as DNAWorks, NIH, USA). Then, chemical synthesis of oligonucleotides with a high error rate was carried out under specific conditions. Four reaction conditions (M1, M3, M5, and M6) were tested for epADS with a Dr. oligo192 platform (BIOLYTIC, US) via classic oligonucleotide synthesis procedures according to manufacturer’s instructions except for the altered reaction conditions. For M1 test, standard dNTPs monomers (DMT-dA(bz) Phosphoramidite, DMT-dG(ib) Phosphoramidite, DMT-dC(bz) Phosphoramidite and DMT-dT Phosphoramidite from Hongen Biotech, Shanghai, China) and fresh DNA synthesis reagents and solvents (ETT Activator, Oxidizing reagent, TCA Deblock, Cap B solution and Acetonitrile, from Hebei DNAchem Biotech, Hebei, China) were used in oligonucleotides synthesis with a 50% reduction in coupling reaction time of standard synthesis program. For the M3 test, standard dNTPs monomers (Hongen Biotech, Shanghai, China) and stored DNA synthesis solvents (Acetonitrile, from Hebei DNAchem Biotech, Hebei, China) were used in oligonucleotides synthesis with a 50% reduction in coupling reaction time of standard synthesis program. In test M5, standard dNTPs monomers (Hongen Biotech, Shanghai, China or Sigma Aldrich, Missouri, USA) and stored DNA synthesis solvents (Acetonitrile, from Hebei DNAchem Biotech, Hebei, China) were used in oligonucleotides synthesis with a 50% reduction in coupling reaction time and deletion of the “Hi drain” step after coupling reaction of standard synthesis program. In test M6, premixed dNTPs* monomers (for all four dNTPs, each contained 99.0% of the main dNTP component along with 0.33% of every other three dNTPs monomer, Hongen Biotech or Sigma Aldrich) and stored DNA synthesis solvents (Acetonitrile, from Hebei DNAchem Biotech) were used in oligonucleotide synthesis with a 50% reduction in coupling reaction time and deletion of the “Hi drain” step after coupling reaction of standard synthesis program. After completion of the chemical synthesis process, the products were quantified and diluted into a working solution of 10 mM. Then the synthetic oligonucleotides were assembled into the target DNA fragment by annealing or PCR. For Blc3 and Blc4 DNA fragments generated by epADS, two long oligonucleotides pairs were assembled by annealing reaction. 10 μl of each oligonucleotide and 10 μl water were mixed up in a PCR tube. Incubated the mixture at 98 °C for 15 min in a thermocycler, and then turned-off the thermocycler to cool down to room temperature (~30 min). The product obtained was used for downstream integration into suitable vectors. For longer DNA fragment (200~1 Kb), synthetic oligonucleotides were assembled by PCR. Briefly, set up the reaction vials as follows: 5 μl Q5® Reaction Buffer (5×); 5 μl dNTP (2.5 mM each); 1 μl of each synthetic oligonucleotide of the target DNA fragment or blocks; 1 μl Q5® High-Fidelity DNA Polymerase; add water to a total volume of 50 μl. PCR program was set as follows: 95 °C, 3 min; 95 °C, 30 s; 63 °C (with a stepwise decrease of 1 °C after each cycle), 30 s; 72 °C, 1 min; recycling 8 cycles; 95 °C, 30 s; 58 °C, 30 s; 72 °C, 1 min; recycling 20 cycles; 72 °C, 10 min; 4 °C, forever. 2 μl PCR products were used as template for the second round of PCR amplification with 1 μl of the first and last oligonucleotides as primers. The second PCR program was set as follows: 95 °C, 3 min; 95 °C, 30 s; 58 °C, 30 s; 72 °C, 1 min; recycling 30 cycles; 72 °C, 10 min, 4 °C, forever. The second round of PCR products was purified by gel eletrophoresis and the DNA band of the right size was cut and transferred to a new 1.5 ml Eppendorf tube. The DNA product generated by epADS was then extracted with a Spin column gel DNA extraction Kit, quantified, and prepared for downstream application.

The comparative study of in vitro generation of genetic diversity by ep-PCR was carried out with a gene encoding fluorescent protein mCherry as those described by the producer. The DNA sequence of the gene encoding fluorescent protein mCherry was listed in Supplementary Data 1. The template plasmid DNA used for ep-PCR which contains the right DNA sequence was produced by normal artificial DNA synthesis process. Two independent ep-PCR experiments with different amounts of template DNA (~50 ng plasmid DNA for Exp.1 and ~5 ng plasmid DNA for Exp.2) were carried out simultaneously. Briefly, set up the reaction vials as follows: 5 μl RandomMut buffer (10×); 5 μl Mutation enhancer (10×); 5 μl dNTP (2.5 mM each); 1 μl (Exp.1) or 0.1 μl (Exp.2) template plasmid DNA; 1 μl forward and reverse primers; 1 μl RandomMut DNA polymerase; add water to a total volume of 50 μl. PCR programs was set up as follows: 94 °C, 3 min; 94 °C, 30 s; 58 °C, 30 s; 72 °C, 1 min; recycling 30 cycles; 72 °C, 10 min, 4 °C, forever. The PCR products were purified by gel eletrophoresis and the DNA band of the right size was cut and transferred to a new 1.5 ml Eppendorf tube. Then the DNA product was extracted with a Spin Column DNA Gel Extraction Kit according to the manufacturer’s instructions. The purified DNA was quantified and used for blunt-end ligation into the pEASY®-T5 Zero Cloning vector as described by the manufacturer. Briefly, 3 µl of inserts and 1 µl of pEASY®-T5 Zero Cloning reagent were mixed up in 200 µl PCR tubes and incubated at room temperature (25 °C) for 10 min, then the mixtures were transformed into DH5a competent cells via standard chemical transformation protocol. Positive colony growth on the LB plates with kanamycin antibiotics was used for mutation profile analysis.

### Plasmids construction and mutation analysis

Plasmids construction was carried out via blunt-end ligation-based cloning (with pEASY®-Blunt Zero Cloning Kit) or seamless DNA assembly respectively. DNA sequences used in this work were listed in Supplementary Data 1. Oligonucleotides sequences used for gene construction were *in silicon* designed with DNAWorks (NIH, USA) and commercially synthesized in Beijing Tsingke Biotech Co., Ltd. (Beijing, China). For cloning of single fluorescent protein-coding genes, the synthesized oligonucleotides were assembled via polymerase chain assembly (PCA) to full-length gene fragments and then ligated into pEASY®-Blunt Zero Cloning vectors as those described by the producer. Briefly, 2 µl of inserts and 1 µl of pEASY®-Blunt Zero Cloning reagent were mixed up in 200 µl PCR tubes, and incubated at room temperature (25 °C) for 10 min. Then the mixtures were transformed into DH5a-competent cells via standard chemical transformation protocol. To construct multi-gene synthetic gene circuits (P1~P6), oligonucleotides were assembled into DNA fragments with predesigned overlaps and then integrated into the vector fragment via seamless DNA assembly as those described by the producer. Briefly, PCR amplification of template plasmid pET28a (+) with specific primers was carried out to obtain the vector fragment. Then set up the reaction vials as follows: 5 μl Q5® Reaction Buffer (5×); 5 μl dNTP (2.5 mM each); 0.5 μl template plasmid DNA; 1 μl forward and reverse primers; 1 μl Q5® High-Fidelity DNA Polymerase; add water to a total volume of 50 μl. PCR programs was set as follow: 95 °C, 3 min; 95 °C, 30 s; 58 °C, 30 s; 72 °C, 3 min 30 s; recycling 30 cycles; 72 °C, 10 min; 4 °C, forever. The PCR products were purified by gel eletrophoresis and the DNA band of the right size was cut and transferred to a new 1.5 ml Eppendorf tube. Then the DNA product was extracted with a Spin Column DNA Gel Extraction Kit according to the manufacturer’s instructions. The obtained DNA fragment was used for DpnI digestion at 37 °C for 30 min to remove residual plasmid DNA. The digested vector fragments were purified with a DNA Extraction Kit and quantified (~50 ng μl^−^^1^) for seamless assembly. 3~4 μl insert DNA fragment generated by epADS, 1~2 μl vector DNA fragment were mixed with 5 μl of seamless assembly reagent and incubated at 50 °C for 30 min. The assembly reaction mixtures were transformed into DH5a competent cells via standard chemical transformation protocol. Transformed cells were plated on LB agar plates supplemented with 50 µg ml^−^^1^ of kanamycin and incubated at 37 °C overnight. Positive colonies were picked and cultured in test tubes or 48-well plates to prepare the plasmids. Mutations in synthesized genes were identified by Sanger sequencing of the prepared plasmids.

### Mutant library construction of multi-gene synthetic gene circuits

P6-*P*_*trc*_*M* mutant library was constructed by replacement of the wild-type sequence in P6 by *P*_*trc*_ DNA fragment generated by epADS. The same strategy has also been used to construct the P1-Blc3M and P1-Blc4M mutant libraries. The DNA fragment generated by epADS was integrated into the vector sequence via seamless assembly as described in “Plasmids construction and mutation” section. The assembly mixtures were transformed into competent cells and plated on an LB plate with kanamycin (50 µg ml^−^^1^). Iterative gene replacement was used to construct the multi-gene synthetic gene circuit mutant libraries P5M6-Run3 and P6M6-Run3. During the first step, wild-type EmGFP genes in plasmid P5 and P6 were replaced with EmGFP DNA fragments obtained through epADS to construct the first round of mutation library (P5M6-Run1 and P6M6-Run1). Then plasmid mixtures of the first round of mutation library were used as template vectors for the second round of recombination. By replacing the wild-type mCherry-coding gene in the template vector obtained from the first round of mutation with mCherry-coding DNA fragments obtained through epADS, we got the second round of mutation library (P5M6-Run2 and P6M6-Run2). With this same strategy, finally, we obtained the multi-gene synthetic gene circuit mutant libraries (P5M6-Run3 and P6M6-Run3). After the third round of gene replacement with the fluorescent gene mutants derived from epADS, transformants were plated on LB agar plates and cultivated overnight at 37 °C to obtain the mutant libraries.

### Phenotype analysis of genetic parts encoding fluorescent proteins, regulatory genetic parts, and synthetic gene circuits generated by epADS

We adopted both fluorescent spectrum and endpoint analysis to characterize the phenotype of cloned genetic parts encoding fluorescent proteins, regulatory genetic parts and synthetic gene circuits generated by epADS. Positive colonies were picked and inoculated into test tubes or 48-well plates filled with 3~4 ml LB medium with appropriate antibiotics and cultured overnight at 37 °C. Fluorescence spectrum of test samples was analyzed with a Hitachi F-7000 Fluorescence Spectrophotometer (Hitachi, Japan). The emission spectrum under specific excitation wavelength (Ex 488 nm for EmGFP, Ex 587 nm for mCherry, and Ex 540 nm for mBanana) of each fluorescent protein was monitored. For endpoint analysis of each fluorescent protein, 50 µl of overnight cultures were transferred to 96-well plates of clear bottom and black side and diluted 4 times into 200 µl. Samples were analyzed with a Biotek 3000 microplate reader (Agilent, USA) following the manufacturer’s instructions. OD_600 nm_ of test samples was also determined for the evaluation of normalized fluorescent strength.

### Combined application of epADS with ALE

A combined application of epADS with ALE was carried out by using the mutant library constructed via epADS as the starting material. Firstly, the target gene sequence (*bla* encoding beta-lactamase in this work) was obtained from NCBI, and primers used for gene synthesis were designed via the online software DNAWorks. Secondly, primers used for *bla* gene synthesis were obtained through epADS under M6 experiment conditions. Thirdly, the synthesized oligonucleotides were assembled into *bla* gene fragments via PCA and inserted into a modified p15C vector (p15C-M1-37-Kan) to replace its native wild-type *bla* gene. The constructed plasmid library was then transformed into *E. coli* DH5a competent cells to obtain the starting mutant library for ALE (designed as AmpM6-R0). Four parallel transformation reactions were carried out simultaneously via normal chemical transformation procedures. After resuspension cultivation, one transformation culture was plated on LB plate with kanamycin resistance and incubated overnight at 37 °C, while the other three transformation cultures were transferred into three tubes filled with 5 ml LB medium (containing 50 µg ml^−^^1^ of kanamycin and 25 µg ml^−^^1^ of carbenicillin) separately to start ALE. All inoculated tubes were incubated at a 37 °C shaker with a rotation speed of 220 rpm for about 12 h, and then 1 ml of culture was retransferred to new tubes filled with 5 ml LB medium containing elevated concentration of carbenicillin. The ALE was carried on for 2 weeks and the concentration of carbenicillin increased from 25 µg ml^−^^1^ to 50 mg ml^−^^1^, while the concentration of kanamycin was kept constant during the ALE process. After 2 weeks of ALE, evolved cultures were sprayed on LB plates containing 5 mg ml^−^^1^ or 10 mg ml^−^^1^ of carbenicillin. Colonies grown on these plates were picked and inoculated into 48-well plates filled with 3 ml LB medium containing 50 µg ml^−^^1^ of kanamycin and 10 mg ml^−^^1^ of carbenicillin. The top 30 cultures with the highest cell density were picked and inoculated separately into tubes filled with 3 ml LB medium containing 50 µg ml^−^^1^ of kanamycin and a very high concentration of carbenicillin (50 mg ml^−^^1^). Cultures growth under this high concentration of carbenicillin was stocked under −80 °C and then used for further comparative growth study with wild-type strain. Plasmids from these cultures were extracted for sequencing to identify possible mutations generated during this process. Transformants of the starting mutant library growth on LB plates (AmpM6-R0) were also picked and cultured for plasmids extraction to determine the mutations introduced by epADS.

### Statistics and reproducibility

A descriptive study was used for the characterization of the mutation profile and phenotype of test genetic parts and synthetic gene circuits generated by epADS under specific conditions, positive colony growth on each transformation experiment (from 10 to ~192 colonies) was all used for mutational and phenotype data analysis. Six biological replicates of each test sample were assayed in the regulation strength test of tryptophan riboswitches generated by epADS, the median value and box plot were used to reflect the difference between different mutations generated. Four biological replicates were conducted for the growth inhibition test of the evolved strain generated by epADS and ALE, and data were shown in a dot plot.

### Reporting summary

Further information on research design is available in the Nature Portfolio Reporting Summary linked to this article.

### Supplementary information

Supplementary Information

Description of Additional Supplementary Files

Supplementary Data 1

Supplementary Data 2

Supplementary Data 3

Supplementary Data 4

Supplementary Data 5

Reporting Summary

## Data Availability

All data of this work are available within this paper and the Supplementary Information Files, while source data for charts/graphs can be found in Supplementary Data 5.
