# Supplementary material for: In vitro generation of genetic diversity for directed evolution by error-prone artificial DNA synthesis
Source: Commun Biol. 2024 May 24;7:628. doi: 10.1038/s42003-024-06340-0 (PMC11126579; doi:10.1038/s42003-024-06340-0)

Supplementary Figure 1. Fluorescent spectrum analysis of the RGB- bio-palette system-P1.

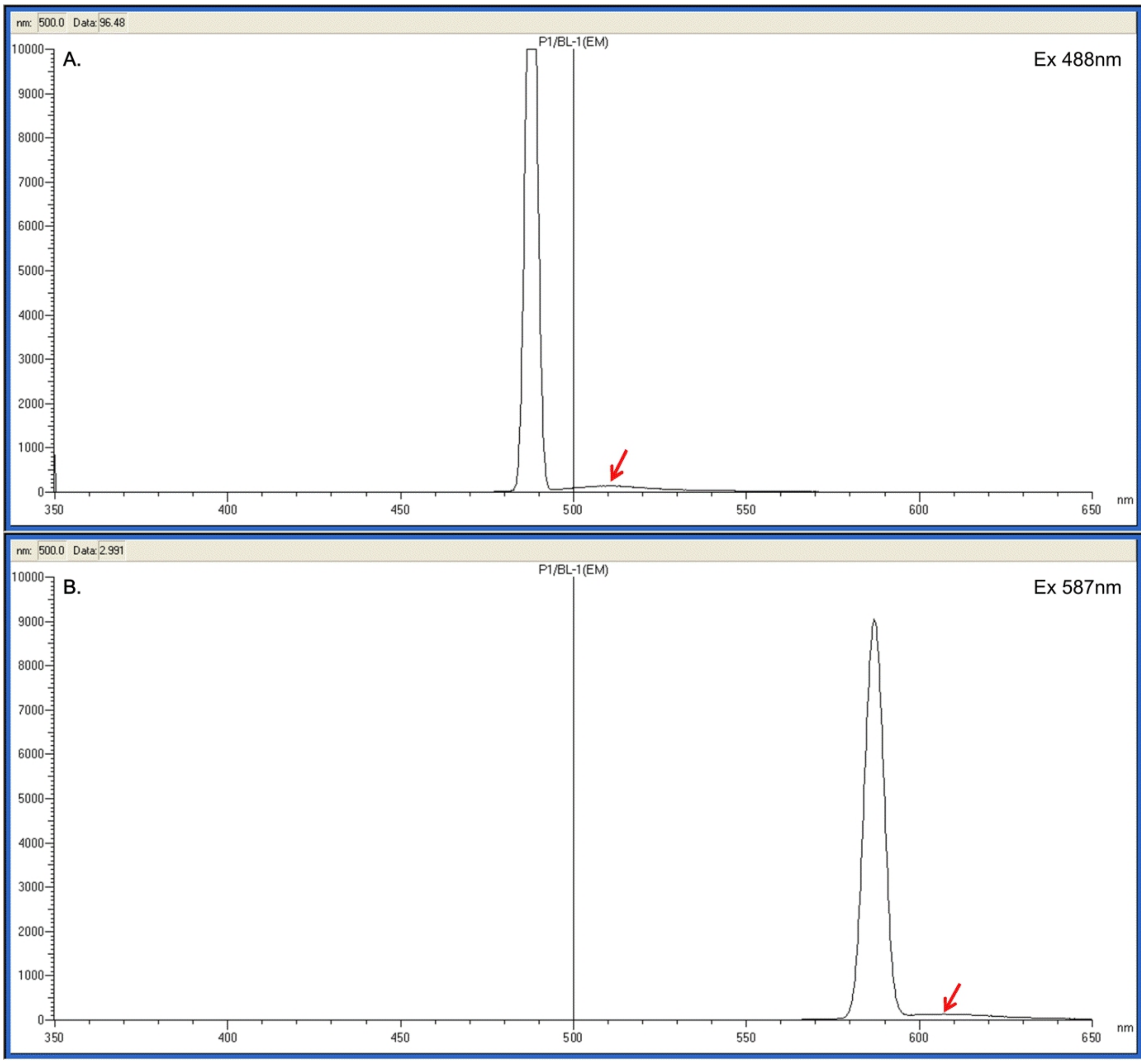

Supplementary Figure 2. Fluorescent spectrum analysis of the RGB- bio-palette system-P2.

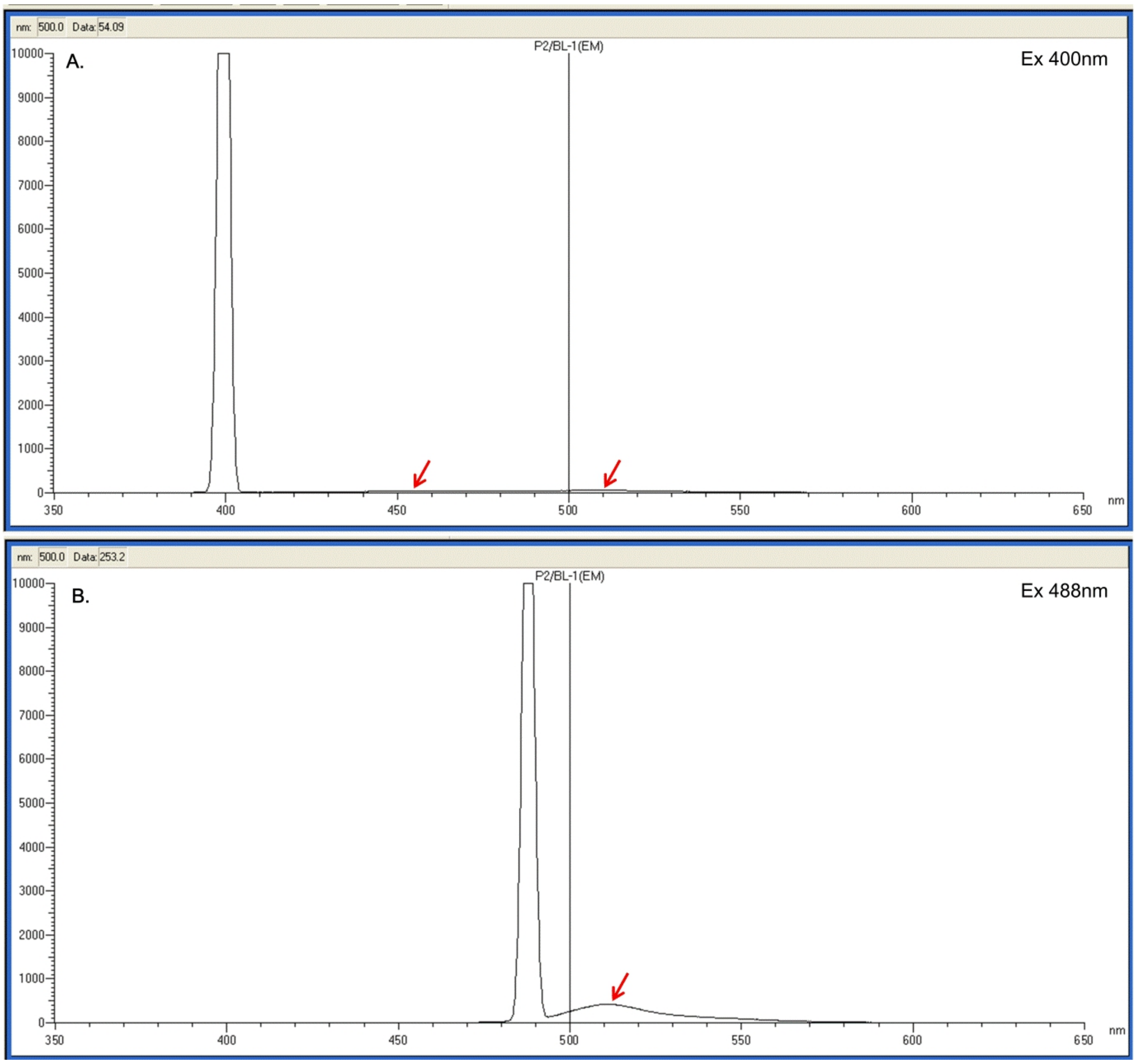

Supplementary Figure 3. Fluorescent spectrum analysis of the RGB- bio-palette system-P3.

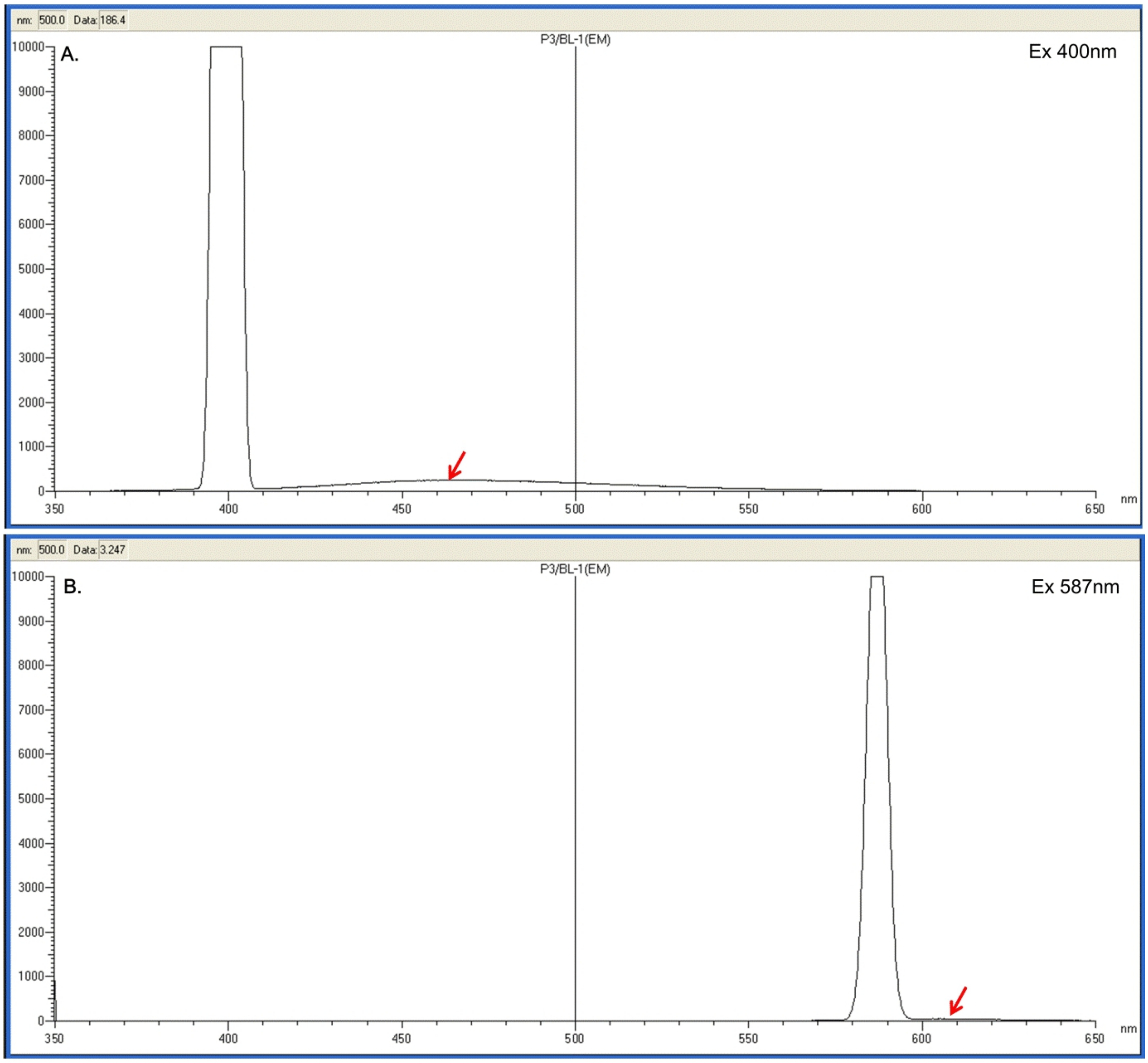

Supplementary Figure 4. Fluorescent spectrum analysis of the RGB- bio-palette system-P4.

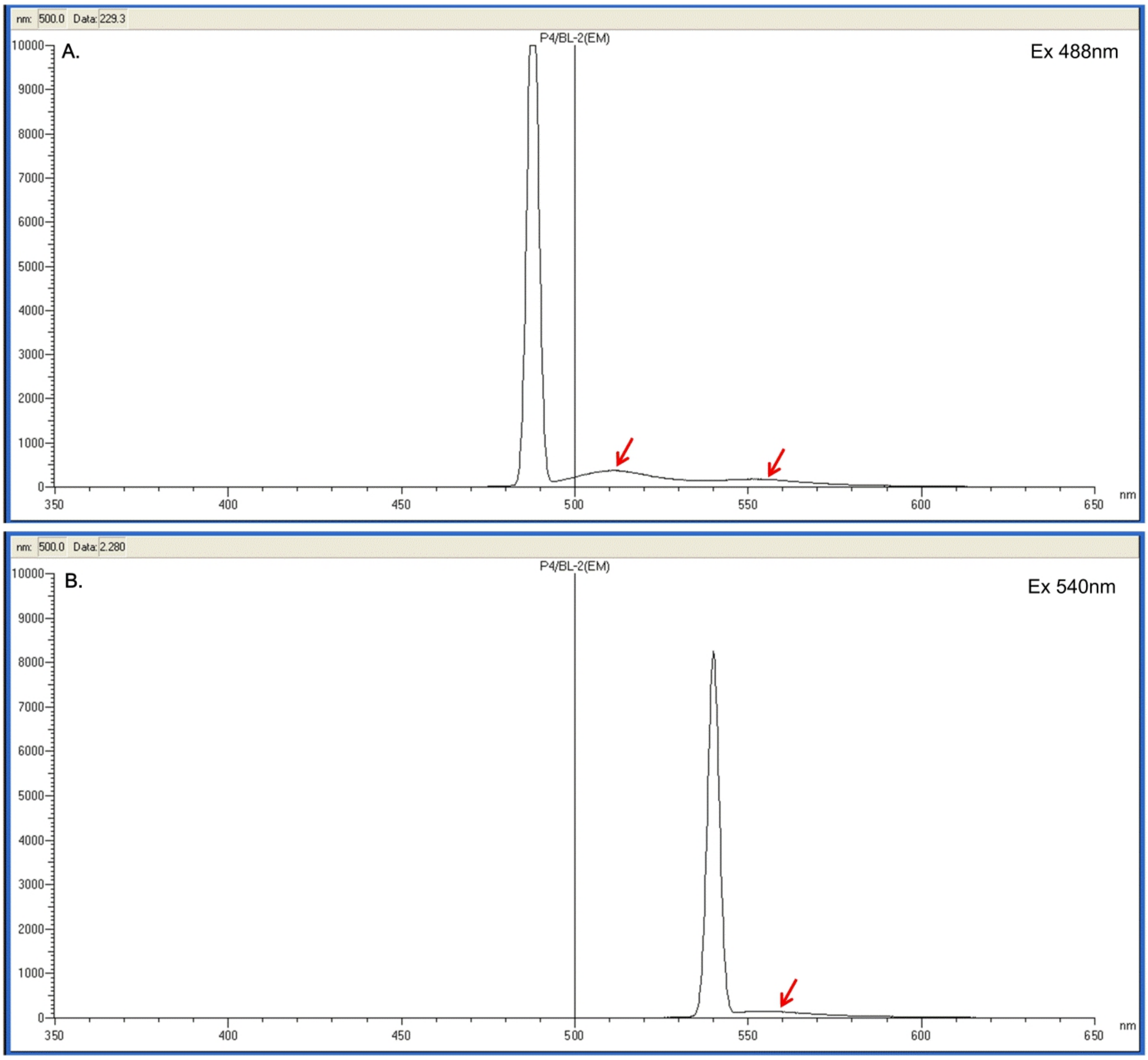

Supplementary Figure 5. Fluorescent spectrum analysis of the RGB- bio-palette system-P5&P6.

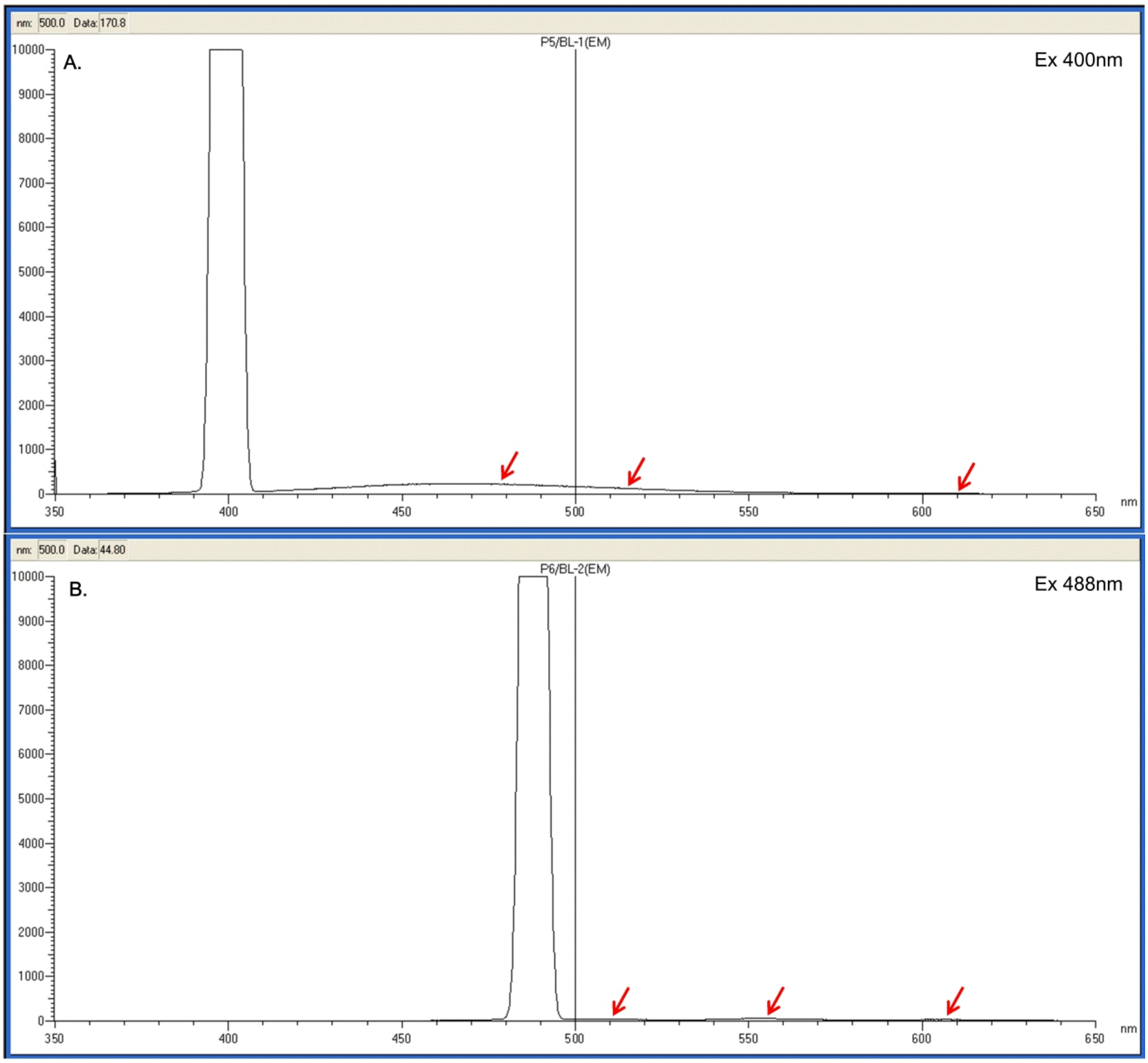

Supplement: Supplementary file 1 — Supplementary Information [file 42003_2024_6340_MOESM1_ESM.pdf]
