# Supplementary material for: In vitro generation of genetic diversity for directed evolution by error-prone artificial DNA synthesis
Source: Commun Biol. 2024 May 24;7:628. doi: 10.1038/s42003-024-06340-0 (PMC11126579; doi:10.1038/s42003-024-06340-0)
Supplement: Supplementary file 2 — Description of Additional Supplementary Files [file 42003_2024_6340_MOESM2_ESM.pdf]

Description of Additional Supplementary Files

File name: Supplementary Data 1

Description: DNA sequences used in this work.

File name: Supplementary Data 2

Description: Mutations generated in the gene encoding mCherry by ep-PCR.

File name: Supplementary Data 3

Description: Mutations identified in bla gene from starting culture (AmpM6-R0/DH5a).

File name: Supplementary Data 4

Description: Mutations identified in bla gene from evolved cultures (AmpM6-R15/DH5a).

File name: Supplementary Data 5

Description: The source data behind the graphs in the paper
