# Supplementary material for: In vitro generation of genetic diversity for directed evolution by error-prone artificial DNA synthesis
Source: Commun Biol. 2024 May 24;7:628. doi: 10.1038/s42003-024-06340-0 (PMC11126579; doi:10.1038/s42003-024-06340-0)
Supplement: Supplementary file 8 — Reporting Summary [file 42003_2024_6340_MOESM8_ESM.pdf]

nature portfolio

Corresponding author(s): Baowei Wang

Last updated by author(s): May 2, 2024

Reporting Summary

Nature Portfolio wishes to improve the reproducibility of the work that we publish. This form provides structure for consistency and transparency in reporting. For further information on Nature Portfolio policies, see our Editorial Policies and the Editorial Policy Checklist.

Statistics

For all statistical analyses, confirm that the following items are present in the figure legend, table legend, main text, or Methods section.

n/a	Confirmed	
☐	☒	The exact sample size (*n*) for each experimental group/condition, given as a discrete number and unit of measurement
☐	☒	A statement on whether measurements were taken from distinct samples or whether the same sample was measured repeatedly
☒	☐	The statistical test(s) used AND whether they are one- or two-sided<br>*Only common tests should be described solely by name; describe more complex techniques in the Methods section.*
☒	☐	A description of all covariates tested
☒	☐	A description of any assumptions or corrections, such as tests of normality and adjustment for multiple comparisons
☐	☒	A full description of the statistical parameters including central tendency (e.g. means) or other basic estimates (e.g. regression coefficient) AND variation (e.g. standard deviation) or associated estimates of uncertainty (e.g. confidence intervals)
☒	☐	For null hypothesis testing, the test statistic (e.g. *F*, *t*, *r*) with confidence intervals, effect sizes, degrees of freedom and *P* value noted<br>*Give P values as exact values whenever suitable.*
☒	☐	For Bayesian analysis, information on the choice of priors and Markov chain Monte Carlo settings
☒	☐	For hierarchical and complex designs, identification of the appropriate level for tests and full reporting of outcomes
☒	☐	Estimates of effect sizes (e.g. Cohen's *d*, Pearson's *r*), indicating how they were calculated

Our web collection on statistics for biologists contains articles on many of the points above.

Software and code

Policy information about availability of computer code

Data collection	Fluorescent spectrum data was collected via FL Solutions 2.1 for F-7000 along with a HITACHI Fluorescence Spectrophotometer, while endpoint analysis and biomass data were collected via Gen5 along with the Biotek synergy neo2 multi-mode reader.
Data analysis	Data analysis and some plotting work were carried out with Microsoft Excel 2020 and open-source software Veusa 3.6.2.

For manuscripts utilizing custom algorithms or software that are central to the research but not yet described in published literature, software must be made available to editors and reviewers. We strongly encourage code deposition in a community repository (e.g. GitHub). See the Nature Portfolio guidelines for submitting code & software for further information.

Data

Policy information about availability of data

All manuscripts must include a data availability statement. This statement should provide the following information, where applicable:

- Accession codes, unique identifiers, or web links for publicly available datasets
- A description of any restrictions on data availability
- For clinical datasets or third party data, please ensure that the statement adheres to our policy

All data of this work were available within this paper and the Supplementary Information Files.

Research involving human participants, their data, or biological material

Policy information about studies with human participants or human data. See also policy information about sex, gender (identity/presentation), and sexual orientation and race, ethnicity and racism.

Reporting on sex and gender	This information has not been collected.
Reporting on race, ethnicity, or other socially relevant groupings	This information has not been collected.
Population characteristics	See above.
Recruitment	This information has not been collected.
Ethics oversight	This information has not been collected.

Note that full information on the approval of the study protocol must also be provided in the manuscript.

Field-specific reporting

Please select the one below that is the best fit for your research. If you are not sure, read the appropriate sections before making your selection.

☒ Life sciences ☐ Behavioural & social sciences ☐ Ecological, evolutionary & environmental sciences

For a reference copy of the document with all sections, see nature.com/documents/nr-reporting-summary-flat.pdf

Life sciences study design

All studies must disclose on these points even when the disclosure is negative.

Sample size	No sample-size calculation was performed. The actual sample size were based on the transformed colonies growth on the plates in mutation frequency analysis. As the number of colonies could not be determined before the transformation experiment, all the colonies growth on these experiments could reflect the actual state of that batch of tests.
Data exclusions	No data exclusion was performed in all this test.
Replication	For phenotype characterization tests of epADS-derived riboswitches and growth test in the combined application of epADS and ALE, three independent biological replicates were carried out simutaneously.
Randomization	This is not relevant to this study. In mutation frequency analysis, all transformed colonies were sequenced for mutation analysis. For phenotype analysis of epADS-derived genetic parts, also all growth colonies were assayed for their effects, so randomization is not relevant to this study.
Blinding	Blinding was not relevant to this study, as no profit or harmful outcomes could be generated to the researchers or potential readers of this study, and biased prejudgment is conflicted with the scientific ethics.

Reporting for specific materials, systems and methods

We require information from authors about some types of materials, experimental systems and methods used in many studies. Here, indicate whether each material, system or method listed is relevant to your study. If you are not sure if a list item applies to your research, read the appropriate section before selecting a response.

Materials & experimental systems

n/a	Involved in the study	
☒	☐	Antibodies
☒	☐	Eukaryotic cell lines
☒	☐	Palaeontology and archaeology
☒	☐	Animals and other organisms
☒	☐	Clinical data
☒	☐	Dual use research of concern
☒	☐	Plants

Methods

n/a	Involved in the study	
☒	☐	ChIP-seq
☒	☐	Flow cytometry
☒	☐	MRI-based neuroimaging

Plants

Seed stocks	This is not relevant to this study.
Novel plant genotypes	This is not relevant to this study.
Authentication	This is not relevant to this study.
